# An experimental approach probing the conformational transitions and energy landscape of antibodies: a glimmer of hope for reviving lost therapeutic candidates using ionic liquid†

**DOI:** 10.1039/d1sc02520a

**Published:** 2021-06-22

**Authors:** Talia A. Shmool, Laura K. Martin, Liem Bui-Le, Ignacio Moya-Ramirez, Pavlos Kotidis, Richard P. Matthews, Gerhard A. Venter, Cleo Kontoravdi, Karen M. Polizzi, Jason P. Hallett

**Affiliations:** Department of Chemical Engineering, Imperial College London South Kensington Campus London SW7 2AZ UK j.hallett@imperial.ac.uk +44 (0)20 7594 5388; Department of Engineering Science, University of Oxford Parks Road Oxford OX1 3PJ UK; Scientific Computing Research Unit, Department of Chemistry, University of Cape Town Rondebosch Cape Town 7701 South Africa

## Abstract

Understanding protein folding in different environmental conditions is fundamentally important for predicting protein structures and developing innovative antibody formulations. While the thermodynamics and kinetics of folding and unfolding have been extensively studied by computational methods, experimental methods for determining antibody conformational transition pathways are lacking. Motivated to fill this gap, we prepared a series of unique formulations containing a high concentration of a chimeric immunoglobin G4 (IgG4) antibody with different excipients in the presence and absence of the ionic liquid (IL) choline dihydrogen phosphate. We determined the effects of different excipients and IL on protein thermal and structural stability by performing variable temperature circular dichroism and bio-layer interferometry analyses. To further rationalise the observations of conformational changes with temperature, we carried out molecular dynamics simulations on a single antibody binding fragment from IgG4 in the different formulations, at low and high temperatures. We developed a methodology to study the conformational transitions and associated thermodynamics of biomolecules, and we showed IL-induced conformational transitions. We showed that the increased propensity for conformational change was driven by preferential binding of the dihydrogen phosphate anion to the antibody fragment. Finally, we found that a formulation containing IL with sugar, amino acids and surfactant is a promising candidate for stabilising proteins against conformational destabilisation and aggregation. We hope that ultimately, we can help in the quest to understand the molecular basis of the stability of antibodies and protein misfolding phenomena and offer new candidate formulations with the potential to revive lost therapeutic candidates.

## Abstract 2

Probing the energy landscape and thermodynamics of biomolecules for drug design.

## Graphical abstract

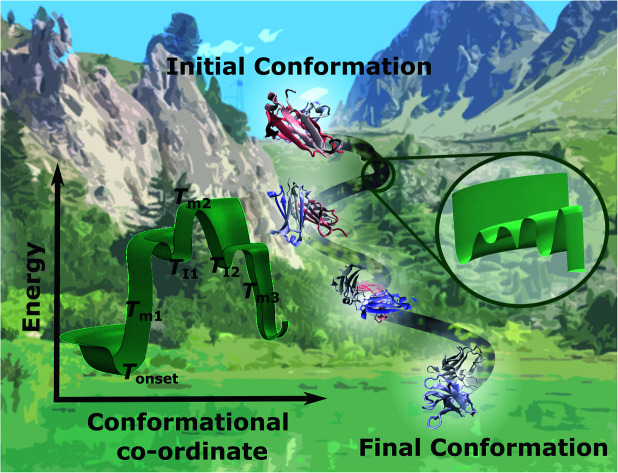

## Introduction

Accumulation of misfolded proteins can cause diseases, including the highly prevalent degenerative diseases Alzheimer's and type II diabetes mellitus.^1^ In a properly folded protein, free energy is minimised by hydrophobic amino acid residues packing together, mostly buried in the protein core, and shielded from water molecules; while hydrophilic residues are exposed to solvent and can interact with water on the protein.^1,2^ Protein misfolding can arise from inherently unstable conformations that can transition from a functional minimum energy state to a new non-functional, potentially toxic state.^3^ For example, new insoluble conformations that form long linear or fibrillar aggregates, known as amyloid deposits, display predominantly β-sheet secondary structure, and are thought to be the cause of Alzheimer's disease.^1,2^ By understanding the mechanism of how such structures arise, the pathway of assembly of intermediate β-sheet conformations and protein folding could advance therapeutic strategies for numerous disease states.

The free energy landscape theory has played a vital role in enhancing our understanding of protein folding and predicting protein structures.^4^ The energy landscape represents the energy of a given protein as a function of all the possible conformations, and underpins molecular events that lead to protein aggregation, encoding the relative stabilities of conformers and the energy barriers between them.^5^ However, due to a lack of appropriate experimental data, the current view of energy landscapes for protein misfolding and aggregation is generally qualitative rather than quantitative.^5,6^ Primarily, computational simulations are used to sample the conformational landscape of proteins.^7,8^ Yet, to date, we are limited by the experimental techniques that can be used for determining the conformational diversity of proteins, particularly since structural characterisation of conformers, other than the most prevalent one, in solution is intensely challenging.^9^ Thus, there is a growing need to develop novel methods to study protein folding, conformational transitions and sample the potential energy landscape of complex systems.

Recently, we presented an original systematic strategy to explore protein conformational space and experimentally detect and characterise ‘invisible’ rare protein conformations using ionic liquids (IL).^10^ ILs are organic salts of relatively high viscosity, and similar to excipient molecules, the interactions of ILs with water and proteins are dominated by hydrogen bonding.^11^ In particular, the use of ILs based on the cationic essential nutrient choline, in combination with a range of biocompatible anions, have raised significant notice for the enhanced stabilisation of different proteins.^10–12^ Choline is attractive as a cation for biocompatible ILs due to its biological origin, and its structure follows the trends for low cation toxicity, with short alkyl chains and a hydroxyl group.^10–13^ Choline-based ILs can include simple, fatty, amino and aromatic organic acids as well as inorganics, with properties such as viscosity, glass transition temperature and thermal stability shown to be highly anion dependent.^12,13^ Additionally, choline-based ILs have been found suitable for protein extraction and purification without degradation or denaturation effects.^14^

When considering thermal denaturation, particularly promising candidates include choline acetate, choline chloride, and choline dihydrogen phosphate ([Cho][DHP]).^15^ These have been shown to increase the thermal stability of α-chymotrypsin, with the stabilising effect of these anions attributed to a change in the water structuring surrounding the protein.^16^ Notably, of the choline-based ILs, [Cho][DHP] has been studied most extensively with a variety of biomacromolecules.^15–17^ For example, the stabilising effects of [Cho][DHP] has been observed on α-chymotrypsin, cyctochrome c and human serum albumin, attributed to the ability to promote protein refolding and resulting in a higher order protein structure.^15–17^ [Cho][DHP] has also been found to significantly stabilise DNA and nuclease and to increase protein thermal and storage stability *via* hydrogen bonding and electrostatic interactions.^15,18^ Thus, we are particularly interested in understanding the effects of biocompatible ILs, specifically [Cho][DHP], on protein stability and the energy landscape and conformational transitions of functional proteins.

By elucidating the conformational transitions and structures of antibodies, we can also advance structure-based drug design. Specifically, over the past five years antibodies have become the best-selling drugs in the pharmaceutical sector,^19^ however, high concentration antibody solutions, deployed in therapeutics, are prone to instability from aggregation and degradation.^20^ Typically, forced degradation studies subject formulations to different stress conditions to estimate the degradation that would occur during a stability study and determine which excipients should be used for enhancing stability and shelf-life.^20,21^ In particular, protein folding and unfolding can be initiated by altering the pH.^10,20–22^ For example, previous work has shown that at low pH monoclonal antibodies can readily aggregate, and hydrophobicity and net charge can account for the distinct aggregation propensity of a given protein at different pH levels.^15,20,22,23^ Therefore, the application of ILs as stabilising agents ought to be carried out with due diligence, in particular when using protic ILs such as [Cho][DHP]. Notably, the possibility of lowering the pH and destabilising the protein can be minimised by using a suitable neutral phosphate buffer.^10^ Additionally, excipients such as sugars, amino acids and surfactants are used to stabilise antibodies in solution.^22–27^ For example, arginine has been found to increase the unfolding transition temperature of immunoglobin G (IgG) in glycine buffer, suppress protein aggregation, and improve refolding of partially folded intermediates.^25^ Similarly, the disaccharide trehalose has been shown to inhibit unfolding and aggregation of the enzyme cutinase following heat shock.^26^ Additionally, the non-ionic surfactant polysorbate 20 was shown to increase the conformational stability of proteins against degradation, inhibiting agitation induced aggregation of recombinant human growth hormone.^27^ Yet, numerous studies have shown that prolonged storage, exposure to temperatures above 50 °C and the presence of water can all reduce the stabilising effects of these conventional excipients.^23,28^ Hence, there is a growing demand for identifying new excipients, predicting and maintaining preferred stable protein conformations.

Given that studying protein conformational diversity is key for understanding protein function, denaturation, misfolding and aggregation, and due to the growing need to develop antibody formulations of enhanced stability, we were motivated to examine the susceptibility of different antibody formulations to stress-induced conformational changes specifically β-sheet transitions in proteins. We hypothesised that changing the temperature and matrix of antibody formulations would enable us to study the conformational transitions, stability against thermal denaturation, and conformational transition pathways of antibodies in different solutions. Concurrently, we hoped to develop innovative high concentration antibody formulations containing ILs for biotechnological applications.

We chose to study human monoclonal antibody immunoglobulin G4 (IgG4), which contains extensive intra- and inter-domain hydrophobic interactions with domains composed primarily of β-sheets and essentially no α-helices.^29^ This is an ideal model system since the denaturation and aggregation of IgG4, which is proposed to be route-dependent, has been studied extensively, with a significant fraction of secondary structure remaining after denaturation induced by heat or low pH.^30^ Recent works examining the structural dynamics of proteins, at different temperatures, for a wide range of protein formulations,^31,32^ revealed two distinct glass transition processes in all formulations. These studies showed that subtle changes in excipient composition affect transition temperatures, and confined proteins may follow different degradation pathways and, or kinetics to a non-confined protein. Extending these works, we have identified and chose to modify two formulation candidates, which showed reduced molecular mobility, directly linked to a decrease in chemical degradation of a material.^31^ We design formulations including the cB72.3 chimeric IgG4, in the absence and presence of 10 wt% [Cho][DHP] with a combination of excipients, including trehalose dihydrate, l-histidine, l-histidine HCl, l-arginine HCl and polysorbate 20. To experimentally monitor temperature and IL-induced conformational transitions of IgG4, we conduct temperature variable circular dichroism (CD) experiments. We determine the thermal and structural stability and changes in β-sheet structure for the antibody systems. We develop a methodology to study the conformational transitions and thermodynamics of the different formulations and explain our observations from the perspective of the energy landscape. Previously, molecular dynamics (MD) simulations have been employed to investigate the preferential inclusion or exclusion of excipients with IgG1, as well as the conformational arrangements of several IgG antibodies.^33,34^ Thus, to gain further insight into the dynamic conformational landscape of the protein, we carry out MD simulations, at low and high temperatures, on a single antigen binding fragment (Fab) of the IgG4 in water, selected excipient solutions, as well as with and without IL. By calculating the theoretical preferential interaction coefficients for each excipient in the different formulations and investigating the localised co-solvent interactions, we determine how protein–excipient and IL interactions shape the conformational landscape of IgG4. Finally, with the ambition to predict and select formulations for drug discovery and early development, we perform bio-layer interferometry (BLI) measurements. By analysing the binding of different Fc and Fab regions to specific antibody-functionalised sensors, we further characterise the formulations and study the combined effect of ILs and excipients on IgG4 structure. Using this combined experimental, analytical and theoretical approach, we aim to explore the conformational transitions of biopharmaceutical systems, deepen our understanding of the molecular basis of protein misfolding, and apply secondary structure element data in drug design.

## Results

### Variable temperature circular dichroism (CD) experiments

**Fig. 1:**
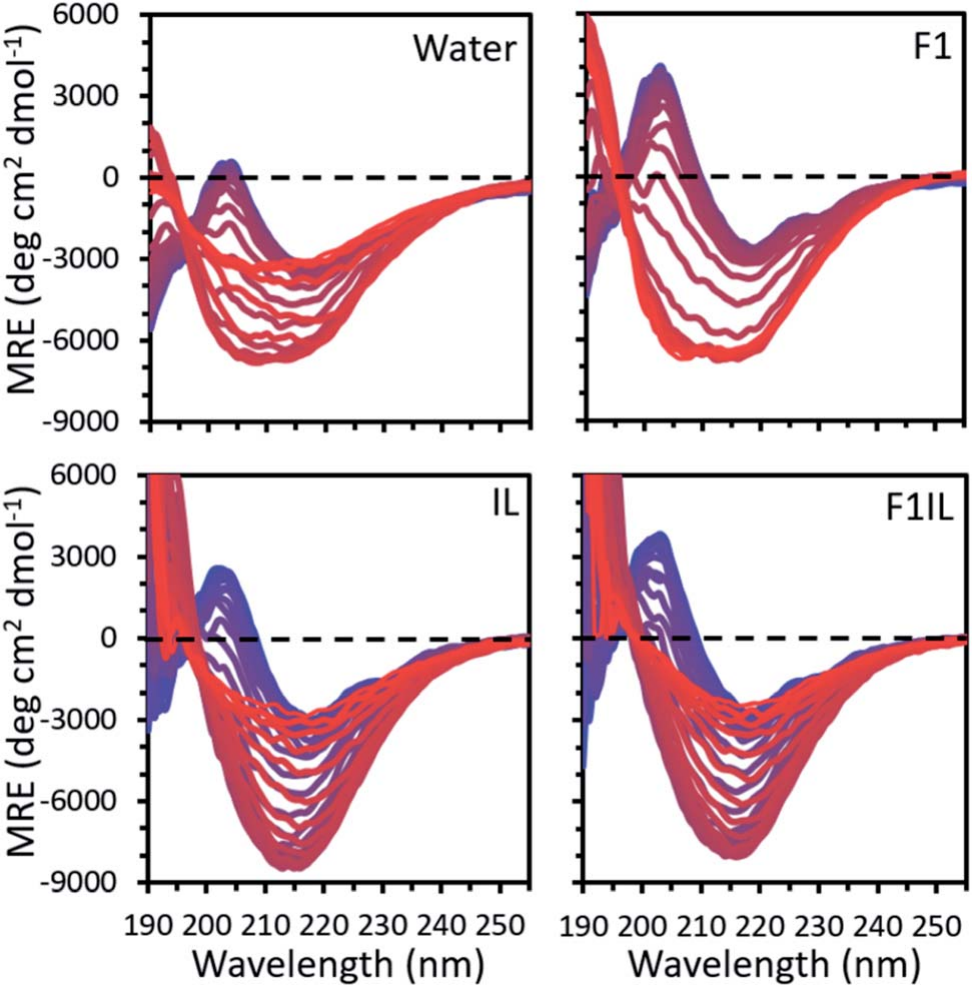
The mean residue ellipticity (MRE) calculated from the temperature variable CD data for IgG4 in pure water, F1, IL and F1IL, measured from 190 to 260 nm with temperature increasing from 25 °C (blue) to 97 °C (red) in 2 °C increments. See Fig. S1† for IgG4 in F2 and F2IL.

Fig. 1 shows the temperature variable CD data of some representative secondary structures found for the formulations examined. For each formulation, our CD spectra qualitatively indicated thermally induced conformational transitions in the antibody secondary structure (Fig. 1 and S1†). We observed a positive peak at 202 nm, attributed to β-turns,^35^ and a strong negative peak at 218 nm, well-defined as indicating β-sheet secondary structure.^36^ An apparent increase in amplitude with temperature in the negative feature is observed, reflecting the thermally induced conformational transitions in β-sheet secondary structure.^36^ For F1 (IgG4, l-arginine HCl, trehalose dihydrate, polysorbate 20) and F2 (IgG4, trehalose dihydrate, polysorbate 20, l-histidine, l-histidine HCl),^32^ we observed a progressive increase in intensity at the characteristic peak at 218 nm with increasing temperature, corresponding to an increase in β-sheet conformations. In contrast, for IgG4 in pure water and the IL containing formulations (IL only, F1IL (IgG4, l-arginine HCl, trehalose dihydrate, polysorbate 20, [Cho][DHP]), F2IL (IgG4, trehalose dihydrate, polysorbate 20, l-histidine, l-histidine HCl, [Cho][DHP])) we observed an increase and subsequent decrease in the intensity of the negative band, corresponding to a loss in the proportion of β-sheet conformations at higher temperatures. Reversibility of the structural changes of IgG4 was examined by cooling F2 and F2IL from 97 °C to 25 °C at 2 °C min^−1^ and measuring the samples at room temperature (Fig. S1†). In both cases, minimal changes in protein structure were observed after cooling, indicating that for these samples, the thermally induced conformational changes are irreversible, in agreement with previous literature.^37,38^ Given that monoclonal antibodies are known to have predominant β-sheet secondary structure,^39^ which is monitored as standard at 218 nm,^40^ and that lower CD wavelengths are associated with more noise,^36^ we selected 218 nm as the wavelength for detailed analysis of the conformational transitions in our systems.

### Finding the conformational transitions and thermodynamics of the formulations

**Fig. 2:**
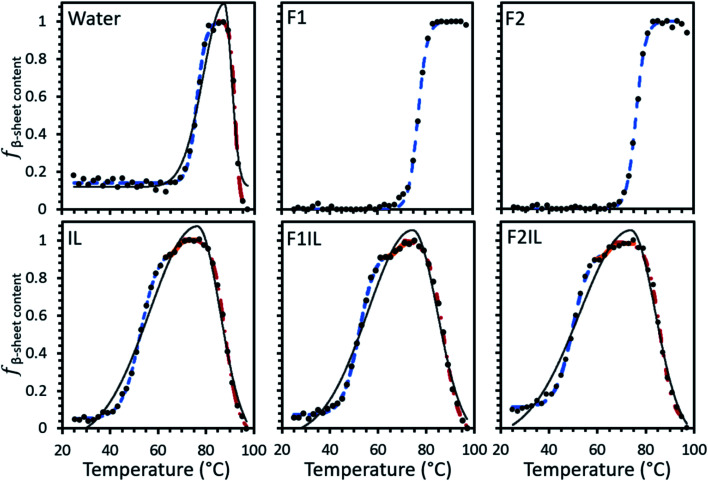
Change in *f*_β-sheet content_, the relative fraction β-sheet content, of IgG4 as a function of temperature in each formulation. *f*_β-sheet content_ was calculated from variable temperature CD signal at 218 nm and scaled linearly so that the conformers with maximum β-sheet content have *f*_β-sheet content_ = 1 and conformers with minimum β-sheet content have *f*_β-sheet content_ = 0. For full details see Materials and methods. Sigmoid 1 (blue dashed line), sigmoid 2 (solid orange line) and sigmoid 3 (red dotted/dashed line).

Using our developed method (see Material and methods for detail), the relative fraction of β-sheet content (*f*_β-sheet content_) was extracted from the MRE data for IgG4 in each formulation, as shown in Fig. 2. The data could be fit by a single sigmoidal curve for F1 and F2, and two distinct sigmoidal regions for IgG4 dissolved in pure water. For the IL containing formulations we could fit three such regions. For IgG4 in water and the IL containing formulations the overall shape of the data could be fit to a bi-Gaussian curve, used here to guide the eye and highlight the difference in behaviour between these formulations and F1 and F2. Despite differences in the overall trend, F1, F2 and water exhibited similar *T*_onset_ values (the onset temperature for conformational transitions), attributed to small-scale conformational changes of the systems.^31,32^ These values were also significantly higher than *T*_onset_ obtained for the IL containing formulations, which also showed two characteristic transition points, *T*_I_1__ and *T*_I_2__, associated with local energy minima separated by barriers, between sigmoidal regions.

In the context of these results, the composition of the excipient matrix and the presence of IL dictated the strength of the conformational transitions, with the number of such changes represented by the number of intersection points (*T*_I_) between adjacent sigmoidal regions (Fig. 2 and Table 1). Table 1 shows that for F1 and F2, the midpoint temperature in the first sigmoidal region (*T*_m1_) was found to be 77.0 °and 76.1 °C, respectively, and 75.7 °C for water. These values were higher than *T*_m1_ of the IL containing formulations (52.1 °C, 52.4 °C and 50.6 °C for IL, F1IL and F2IL, respectively), indicating that the conformers exhibited higher thermostability in the absence of IL. Additionally, *T*_m2_ was higher for water than for the IL containing formulations, with little variation in *T*_m2_ and *T*_m3_ values for the different IL containing formulations. Similarly, *T*_I1_ of water was higher than for the IL containing formulations, with little difference observed for *T*_I1_ or *T*_I2_ between the IL containing formulations. Evidently, for all formulations, upon heating, the antibody in its respective excipient matrix could undergo conformational transitions.^32^

**Table 1:** The temperatures of the sigmoidal midpoints (*T*_m_) and intersections between two sigmoid curves (*T*_I_) for each formulation, calculated from the sigmoid curves fitted in Fig. 2, as described in Materials and methods. *T*_m_1__, *T*_m_2__ and *T*_m_3__ represent the temperatures of the sigmoidal midpoints for region 1, 2, and 3, respectively. *T*_I_1__ and *T*_I_2__ represent the intersection between sigmoidal region 1 and 2, and sigmoidal region 2 and 3, respectively

Formulation	*T*_m_1__ (°C)	*T*_m_2__ (°C)	*T*_m_3__ (°C)	*T*_onset_ (°C)	*T*_I_1__ (°C)	*T*_I_2__ (°C)
Water	75.5 ± 0.1	91.83 ± 0.03	—	72 ± 4	86.3 ± 0.1	—
IL	52.1 ± 0.1	68.8 ± 0.1	88.6 ± 0.1	45 ± 2	63.5 ± 0.1	76.5 ± 0.1
F1	77.0 ± 0.1	—	—	73 ± 5	—	—
F2	76.1 ± 0.1	—	—	72 ± 5	—	—
F1IL	52.4 ± 0.1	67.5 ± 0.4	86.8 ± 0.1	45 ± 2	62.5 ± 0.1	73.5 ± 0.1
F2IL	50.6 ± 0.1	63.3 ± 1.5	86.0 ± 0.1	44 ± 2	59.4 ± 0.1	73.1 ± 0.1

To gain further insight into the conformational transitions in each system, we determined the change in enthalpy (Δ*H*_m_) and entropy (Δ*S*_m_), respectively for each formulation (Table 2). We found that Δ*H*_m_1__ and Δ*S*_m_1__ were highest for the excipient only formulations (F1 and F2), then water, and lower for the IL containing formulations (IL, F1IL, F2IL). This indicated that the presence of excipients raised these values compared to water, while the addition of IL decreased Δ*H*_m_1__ and Δ*S*_m_1__. It is worth noting that Δ*H*_m_1__ and Δ*S*_m_1__ were higher for F1 than F2 (120 kJ mol^−1^, 1560 J K^−1^ mol^−1^ and 104 kJ mol^−1^, 1360 J K^−1^ mol^−1^, respectively); and in the presence of IL these were higher for F2IL than F1IL (53 kJ mol^−1^, 1050 J K^−1^ mol^−1^ and 48 kJ mol^−1^, 910 J K^−1^ mol^−1^, respectively). The Δ*H*_m_2__ of water and IL were comparable, though Δ*S*_m_2__ of IL (3800 J K^−1^ mol^−1^) was much larger than water (3090 J K^−1^ mol^−1^). In F1IL and F2IL both values were much lower (Δ*H*_m_2__ of 150 kJ mol^−1^ and 134 kJ mol^−1^ and Δ*S*_m_2__ of 2200 J K^−1^ mol^−1^ and 2120 J K^−1^ mol^−1^, respectively), and we observed that at *T*_m_3__, the values of Δ*H*_m_3__ and Δ*S*_m_3__ were significantly reduced for all the IL containing formulations, though less so in the case of IL only.

**Table 2:** Thermodynamic values of change in enthalpy (Δ*H*) and entropy (Δ*S*) for the formulations examined, calculated for the different sigmoidal regions of the *f*_β-sheet content_ curves

Formulation	Δ*H*_m_1__ (kJ mol^−1^)	Δ*S*_m_1__ (J K^−1^ mol^−1^)	Δ*H*_m_2__ (kJ mol^−1^)	Δ*S*_m_2__ (J K^−1^ mol^−1^)	Δ*H*_m_3__ (kJ mol^−1^)	Δ*S*_m_3__ (J K^−1^ mol^−1^)
Water	93 ± 7	1230 ± 90	284 ± 8	3090 ± 90	—	—
IL	47 ± 1	910 ± 20	260 ± 20	3800 ± 300	112 ± 2	1290 ± 10
F1	120 ± 10	1560 ± 70	—	—	—	—
F2	104 ± 7	1360 ± 60	—	—	—	—
F1IL	48 ± 2	910 ± 40	150 ± 30	2200 ± 500	81 ± 2	960 ± 10
F2IL	53 ± 1	1050 ± 20	134 ± 4	2120 ± 30	73 ± 4	870 ± 30

While it was not possible to calculate the total change in Gibbs free energy (Δ*G*_total_) for F1 and F2, Table 3 shows that the total change in enthalpy (Δ*H*_total_) and entropy (Δ*S*_total_), respectively, were lowest for F2 and F1 from the formulations examined. Additionally, we found that Δ*G*_total_ was highest for water (2.19 kJ mol^−1^), followed by F1IL, then F2IL, and then IL (−40.5 kJ mol^−1^); yet conversely, Δ*H*_total_ and Δ*S*_total_, were highest for IL, followed by water, F1IL, and F2IL. It is worth noting that while we observed qualitative similarities for water and the IL containing formulations in Fig. 2, the differing energies shown in Tables 2 and 3 appear to indicate that there are multiple pathways for conformational transitions of IgG4.^10^

**Table 3:** Thermodynamic values of total change in enthalpy (Δ*H*_total_), entropy (Δ*S*_total_), and Gibbs free energy (Δ*G*_total_) for the formulations examined

Formulation	Δ*H*_total_ (kJ mol^−1^)	Δ*S*_total_ (J K^−1^ mol^−1^)	Δ*G*_total_ (kJ mol^−1^)
Water	390 ± 20	4500 ± 200	2.19 ± 16.1
IL	420 ± 20	6000 ± 300	−40.5 ± 35
F1	120 ± 10	1560 ± 70	—
F2	104 ± 7	1360 ± 60	—
F1IL	280 ± 30	4100 ± 500	−21.3 ± 45.9
F2IL	260 ± 6	4040 ± 50	−35.1 ± 6.6

**Fig. 3:**
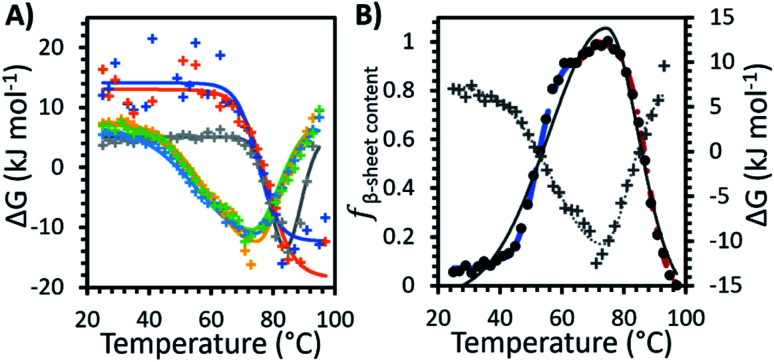
(A) The change in Gibbs free energy (Δ*G*) with temperature for the formulations measured: water (grey), F1 (orange), F2 (dark blue), IL (yellow), F1IL (green) and F2IL (light blue). Solid lines represent the best fit determined systematically for each data set: sigmoid curve for F1 and F2, Gaussian curve for water and bi-Gaussian curve for IL, F1IL and F2IL. (B) The change in Gibbs free energy (grey plus) with bi-Gaussian fit (grey dotted line) and *f*_β-sheet content_, the relative fraction β-sheet content (Fig. 2, black circles with 3 sigmoid fits) with bi-Gaussian fit (black solid line) for F1IL plotted on the same graph.

Fig. 3A shows the Gibbs free energy change (Δ*G*) upon secondary structural changes in the different samples. The curves exhibited a bi-Gaussian shape for the IL containing formulations, a Gaussian shape for water, and a sigmoidal shape for F1 and F2 as a function of temperature (similar to the behaviour we observed for each formulation in Fig. 2). Distinctly, for F1 and F2, which exhibited enhanced thermostability, as indicated by the raised value of *T*_m_1__ (Table 1), Δ*G* decreased steeply with temperature above *T*_onset_ (Fig. 3A) as *f*_β-sheet content_ increased (Fig. 2). This demonstrated that varying structural features, specifically increasing β-sheet content, contributed to enhanced thermostability of the conformers in the absence of IL. Fig. 3A shows that for each formulation above *T*_m_1__, Δ*G* approached a minimum, correlated to the temperature region where the system entropy is maximised, and the conformational states showed the greatest *f*_β-sheet content_ (Fig. 1, 2 and Table 2). Distinctly, for water and the IL containing formulations, with heating above *T*_m_2__, and further above *T*_m_3__, Δ*G* increased continuously with temperature, while *f*_β-sheet content_ decreased gradually. This is exemplified in Fig. 3B for F1IL (see Fig. 2 and 3A for other samples).

### Molecular dynamics simulations

**Fig. 4:**
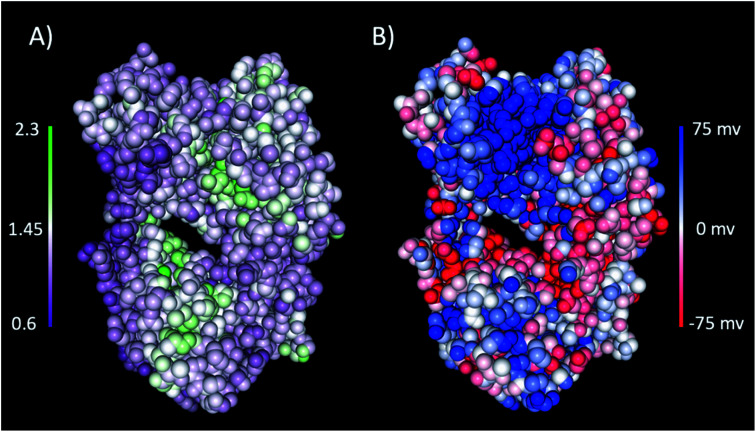
(A) The NPP ratio and (B) surface charge map for the Fab fragment. In (A) the Fab is coloured from low NPP ratio (purple) to high NPP ratio (green), and in (B) the Fab is coloured from negative charge (red) to positive charge (blue). The surfaces were obtained from the PDB structure using the protein-sol patches software.^41^

The interaction of excipients with Fab fragments have been shown to be highly dependent on the surface residues of the Fab fragment.^33,34^ Therefore, we first characterised the Fab fragment in terms of the NPP ratio (ratio of non-polar to polar solvent accessible surface areas) and surface charge. Fig. 4 shows surface plots of NPP ratio and charge mapped on the investigated Fab fragment. Visualisation of the hydrophobicity and charge surfaces reveal that the investigated Fab fragment is predominantly polar in nature and that a high positive charge is localised in the variable region.

To examine the structural stability of the antibody in the different formulations, we simulated the Fab fragment from IgG4 at pH 5.5 at low and high temperatures, for water, IL, F1 and F1IL. Based on the similarity of behaviour and CD spectra of F2 to F1 and F2IL to F1IL, respectively, these formulations were not included for the purpose of the MD simulations. Specifically, MD simulations at high temperatures were performed to investigate the thermal stability of the Fab fragment and the role of excipients and IL on the dynamics of the systems.

**Fig. 5:**
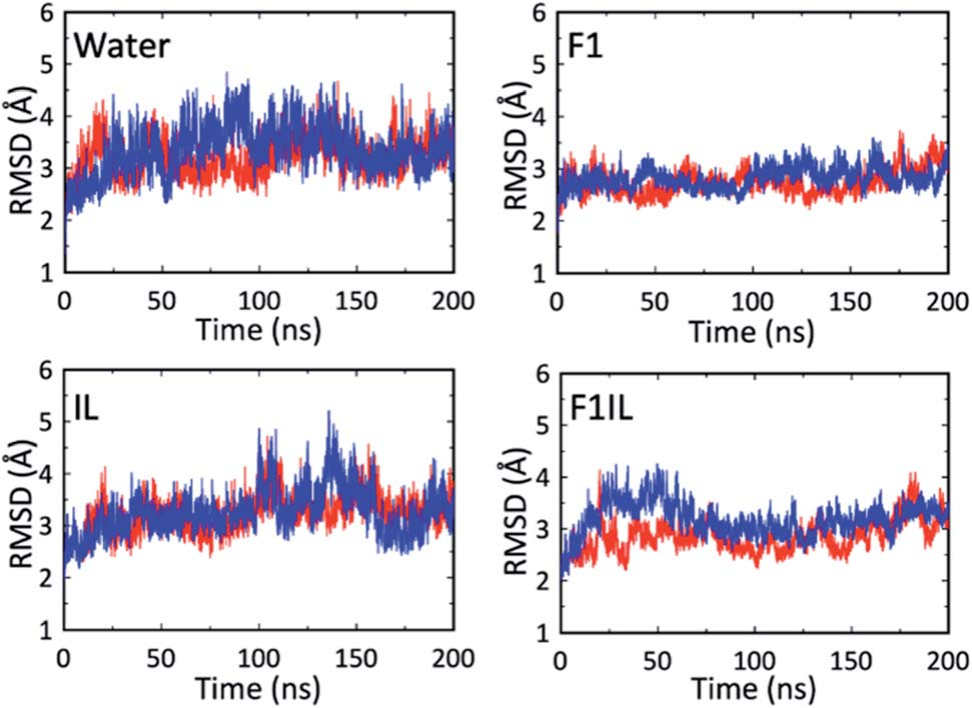
RMSD of the Fab heavy atoms from the X-ray structure during the MD simulations at high temperatures. The red line is for simulation 1 and the blue line is for simulation 2.

Fig. 5 shows the root-mean-square deviations (RMSD) of the protein heavy atoms for each simulation at high temperatures. These plots indicate that the mobility of the Fab fragment was most restricted in F1, and least restricted in the water simulations. In agreement with Fig. 1, 2 and Table 2, for IL and F1IL, the dynamics of the Fab fragment exhibited similar behaviour. Additionally, the RMSD data from the MD simulations at low temperatures (Fig. S5†), showed that the motions of the Fab fragment were restricted in F1, IL and F1IL compared to water. Moreover, we observed that the addition of [Cho][DHP] and arginine HCl, restricted the mobility of the Fab fragment at low temperatures, leading to an increase in effective solvent viscosity. Notably, the increase in RMSD observed for the simulations of both IL and F1IL at high temperatures, indicate that the motions of the Fab fragment and its matrix become less restricted.

To supplement the RMSD analysis, probability distributions of RMSD *vs.* radius of gyration were generated for each of the simulations (Fig. S6†). From the simulations at high temperatures, the 2D contour plots reveal a reduction in the conformational space sampled by the Fab fragment in F1 compared to water, IL and F1IL. Furthermore, for F1 we observe that the conformational space sampled remained low at both temperatures examined, indicating that the excipients in F1 imparted greater stabilisation on the Fab fragment compared to IL and F1IL.

**Fig. 6:**
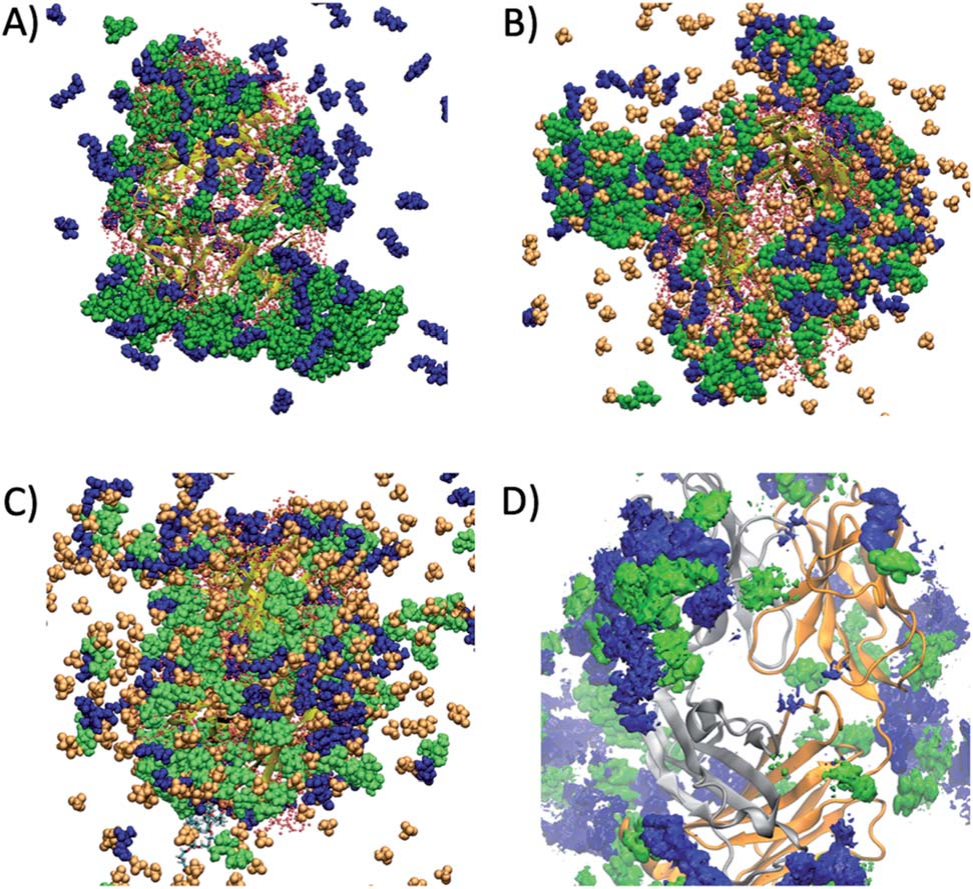
(A) Snapshot of the interaction of trehalose (green) and arginine (blue) molecules around the Fab region in F1 at low temperatures. (B) Snapshot of trehalose (green), arginine (blue) and dihydrogen phosphate molecules around the Fab region in F1IL at low temperatures, and (C) at high temperatures. (D) Spatial distribution function showing the highest probability regions of finding trehalose (green) and arginine (blue) molecules around a Fab fragment in F1IL at high temperatures.

Preferential interaction coefficients, *Γ*_23_ or *Γ*_MD_, were determined for each of the excipients and IL components, in order to gain a deeper insight into their effects on the dynamics and conformational sampling of the Fab fragment. *Γ*_MD_ were calculated at low and high temperatures, to investigate the effect of temperature on protein–excipient/IL interactions. A summary of the preferential interaction coefficients is shown in Table S3,† including the different arginine and choline group combinations considered for F1IL. From the MD simulations, each of the considered excipients, including the IL, were found to preferentially interact with the Fab fragment at both temperatures investigated. Fig. 6 shows a snapshot of the spatial arrangement and interaction sites of excipients and ILs with the Fab fragment in (A) F1 at 27 °C, and (B) F1IL at 27 °C and (C) F1IL at 127 °C. Fig. 6D shows a time-averaged spatial distribution of trehalose (green) and arginine (blue) for the last 50 ns of simulation 1 for F1IL at 127 °C. The MD analysis indicated that protein–excipient interactions were favoured over protein–water interactions. Specifically, for systems containing trehalose (F1 and F1IL), we found that the sugar has a strong affinity for the protein surface. For systems containing ions, we found that the larger, hydrophobic groups (arginine and choline cation, and dihydrogen phosphate anion) bind favourably with the protein surface. In contrast, the chloride anions were preferentially excluded from the protein surface. Moreover, calculated preferential binding coefficients for the two potential dihydrogen phosphate groups of [Cho][DHP] and [Arginine][DHP] (Table S3†), present in IL and F1IL, showed significant preferential binding coefficients at both low and high temperatures. Notably, analysis revealed that excipient–excipient and excipient–IL clusters were formed *via* hydrogen bonds, between trehalose, arginine and dihydrogen phosphate, in the respective formulations. Taken together, these results provide evidence that the dihydrogen phosphate anions destabilise the Fab fragment and facilitate protein dynamics, in agreement with the CD data analysis.

### Bio-layer interferometry (BLI) analysis

**Fig. 7:**
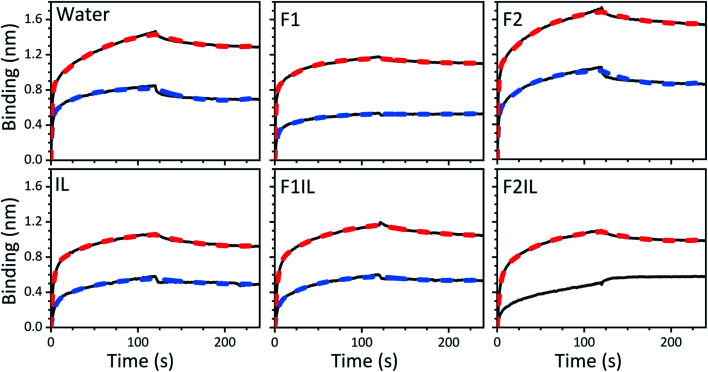
BLI sensorgrams showing the binding of the fragment crystallisable (Fc) region (top black solid line) and the antigen-binding fragment (Fab) region (bottom black solid line) of the IgG4, prepared in water, F1, F2, IL, F1IL and F2IL, respectively. Dashed lines show the 1 : 2 model fit for each experiment (red for Fc and blue for Fab regions, respectively). The sensorgram for the Fab region in F2IL could not be fit appropriately using the 1 : 2 binding model and is thus only qualitatively compared to the other systems examined.

We performed BLI experiments to examine the effects of IL and excipients on IgG4 structure. BLI is ideal for our study as it is a rapid method, easily adaptable to different buffer conditions, requires minimal amounts of sample and permits real-time and label-free analyses. Specifically, we used antibody-functionalised BLI sensors targeting the fragment crystallisable (Fc) and Fab regions of the IgG4 (see Materials and methods for detail). Typically, sensorgrams follow 1 : 1 binding kinetics, where one ligand molecule interacts with one analyte molecule, and the binding is independent and of equal strength for all the analyte–ligand interactions. After the initial baseline measurement during the equilibration step (which was flat, and therefore, omitted from the figure), these sensorgrams show an initial steep increase in the signal, before quickly reaching a plateau. However, in our sensorgrams, the initial steep increase was followed by a second stage with a slower binding rate, attributed to a heterogeneous interaction between the IgG4 and the sensor (Fig. 7). After establishing that a 1 : 1 model did not satisfactorily fit our experimental results, as can be observed in the discrepancies between experimental and fitted data (Fig. S2†), the binding profiles of the IgG4 were fitted to a 1 : 2 binding model (Fig. 7). This approach considered an initial analyte–ligand binding and a posterior formation of a bridged complex, with the analyte binding to a second ligand molecule of the sensor in close proximity to the first. This behaviour is known as avidity, and can be observed for bivalent analytes, such as antibodies.^42,43^ It should be noted that the explanation of avidity only applies to the Fab region of the IgG4, and the secondary binding stage was also observed for the Fc region. Therefore, the contribution of interspecific interactions, and, or IgG4 regional concentration in the vicinity of the sensor should be considered as well. In any case, the constants corresponding to the initial binding, equilibrium (*K*_D_) and association (*k*_on_1__) and dissociation (*k*_off_1__) rate constants (Table S1†), reflect the properties of the antibody–ligand binding and any structural alterations or disruptions of the IgG4, affecting their interaction. We note that the binding behaviour in the second stage was beyond the scope of this work, and further experimentation would be required to elucidate its nature.

A qualitative analysis of the sensorgrams, comparing the slope during the adsorption step, revealed that for F2 the antibody–ligand interaction was stronger compared to the control experiment in water, while it was lower for F1. As expected, in the absence of stabilising excipients, we observed a strong decrease in the BLI signal for IgG4 in IL.^28,44^ This provided additional evidence for IL-induced conformational changes of the antibody. Moreover, the small difference between the sensorgrams of F1 and F1IL highlighted that for F1, the interference of the IL on IgG4 interaction with both sensors was attenuated. In contrast, a considerable decrease in the interaction strength of IgG4 was observed for F2IL compared to F2.

Based on the parameters obtained by fitting the sensorgrams to a 1 : 2 binding kinetic for the formulations examined, we found that the presence of IL resulted in a decrease of the association rate of *k*_on_1__ (Fig. 7 and Table S1†). This was attributed to the IL hindering the formation of the IgG4–ligand complex. Additionally, for F1IL and F2IL the values of *k*_off_1__ were higher compared to F1 and F2, indicating that the IgG4–ligand complex was more prone to dissociation in the presence of IL. This was different from the behaviour observed in water, where the value of *k*_off_1__ showed no change and no decrease was observed for the Fc and Fab regions, respectively. We note that the dissociation rates observed were minimal for all cases. As a consequence of the slight increase in *k*_off_1__ and, or in *k*_on_1__, the value of *K*_D_ was raised in the presence of IL in all cases. Furthermore, the *K*_D_ values found for both the Fab and Fc regions of IgG4 in F1 and F1IL were smaller than their equivalent in water and F2. Notably, in agreement with other IgG4 systems measured by BLI our calculated values were in the range of nm scale.^45,46^

## Discussion

### Navigating the energy landscape of protein formulations

**Fig. 8:**
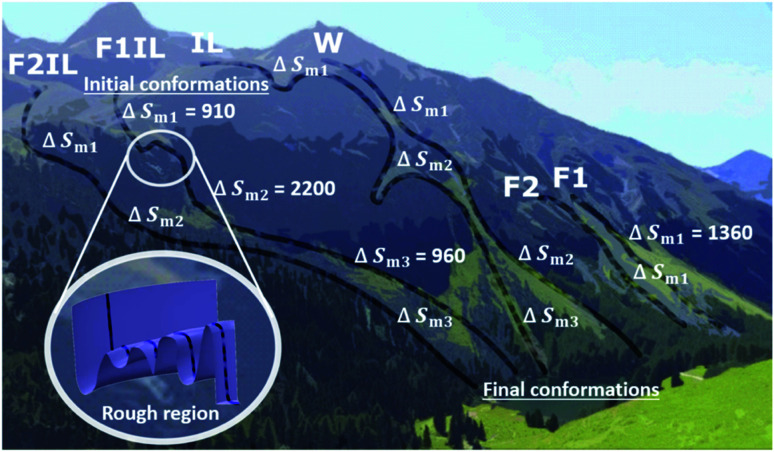
Schematic for the different conformational transition pathway for each system indicated by faint black lines from initial to final conformations (animated photograph of the Swiss Alps taken by T.A.S.). Due to the dynamic ruggedness of the landscape there are many minima of local free energy. For the excipient only formulations, F1 and F2, the pathways go through smooth regions of the free energy landscape. The conformers can roll down different routes toward the final conformations, and when IgG4 and the matrix are confined, compact conformers different from the native, initial conformations are formed. For water and the IL containing formulations, F1IL and F2IL, the pathways go through rough regions of the free energy landscape. The conformers pass through intermediate traps, and are delayed en-route to the bottom, leading to conformational heterogeneity of intermediate states, eventually forming the final conformations.

With the aid of Fig. 8, for each formulation, we can visualise the antibody conformational transitions and pathways. Fig. 8 illustrates that the conformational energy landscape of IgG4 is rugged, with many local minima, similar to a real mountain range, with hills and valleys and smooth surfaces with almost no traps. Under denaturing conditions, we envision that the ensembles of conformers are distributed over the mountain top, and travel along different pathways down the mountain. Applying the energy landscape metaphor, comparing F1 and F1IL in Fig. 8, the conformational ensembles could take a smooth straight path down the mountain, or could enter a rough region and become trapped transiently in the many local energy minima before returning to the downhill search. Taken together with the thermodynamic parameters determined from the CD data and the MD simulations analysis, we established that for the different formulations, a large number of different states would be located on the surface of the mountain, with ensembles demonstrating both low and high conformational entropy (Fig. 8 and Tables 2, 3). Thus, small changes in the matrix could lead to continuous subtle changes in the populations of these molecular conformations.

### The effects of excipients and IL on the conformational transitions and thermodynamics of antibodies

**Fig. 9:**
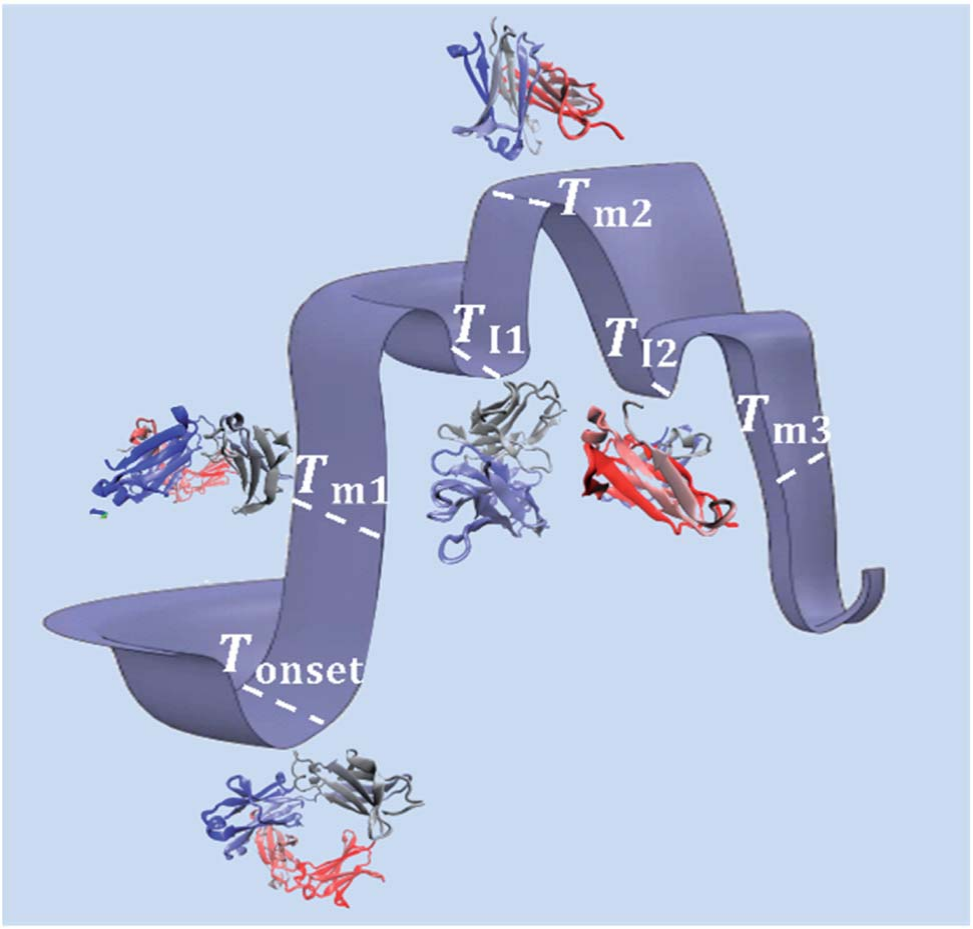
Schematic of the conformational transitions of IgG4 in the IL containing formulations including five representative states. *T*_onset_ indicates the temperature threshold at which the conformers have sufficient free energy to begin exploring various conformational environments (represented by the different three-dimensional structures of the antibody at the marked temperature points).^49,50^ With additional energy input, the conformers can overcome greater energy barriers and achieve larger conformational changes, reflected by the change in secondary structure, specifically the formation of β-sheet rich protein conformations at *T*_m_1__. The conformers can continuously explore an ensemble of conformational changes, through thermal activation, until becoming trapped in free energy minima, represented by *T*_I_1__ and *T*_I_2__, or large-scale conformational changes and trapping lead to unfolding of the conformers at higher temperatures.

As illustrated in Fig. 9, *T*_onset_, *T*_m_ and *T*_I_, respectively, establish a corresponding location along the conformational transition pathway. In F1 and F2, at *T*_onset_ = 73 °C and 72 °C, respectively, the conformers could begin exploring a range of conformations,^47^ and with heating above *T*_m_1__, could overcome higher energy barriers and achieve larger conformational changes, until becoming trapped in an energy minimum that exceeds the available thermal energy.^32,48^ This is represented by the plateau of the sigmoidal curve above approximately 85 °C (Fig. 2). IgG4 in water showed a comparable *T*_onset_ to F1 and F2, with Δ*H*_m_1__ and Δ*S*_m_1__ values that fall between the two; yet we identified two distinct sigmoidal regions. It can be suggested that the propensity of the conformers to undergo conformational changes is reflected by the different sigmoidal regions of *f*_β-sheet content_ as a function of temperature curve (Fig. 2). It appears that for the case of water, the conformers became transiently trapped in a local energy minimum, *T*_I_1__ (Fig. 2 and 9), and with further heating above *T*_I_1__, had sufficient free energy to escape the local energy well, and thereafter the conformers gradually lost secondary structure (specifically β-sheet content decreased with temperature).

Distinctly, each of the IL containing formulations showed a bi-Gaussian temperature dependence, and *T*_onset_ was significantly lower for the IL containing formulations (Table 1), indicating that the presence of IL had a strong effect on the onset temperature for conformational transitions. Correspondingly, the lower Δ*H*_m_1__ observed, with the addition of IL, may indicate that IL molecules resulted in reduced local barriers, allowing for more low temperature conformational transitions. With the aid of Fig. 2 and 9 we can visualise that from room temperature to *T*_I_1__, we observed a similar behaviour to IgG4 in water, yet with further heating above *T*_I_1__ the conformers had sufficient free energy to escape the local energy well, and get easily trapped in another local minimum, *T*_I_2__, corresponding to conformers containing the highest β-sheet content. With additional energy input, the conformers could overcome greater energy barriers and achieve larger conformational changes, reflected by the change in secondary structure (Fig. 1 and 2). These observations suggest that for systems like water and the IL containing formulations, the conformers can easily get trapped in one of the local minima, and as such do not have a unique well-defined conformational state. This is further supported by the CD data and the MD simulations analysis, which demonstrate that the degree of conformational changes of F1, F2, and water was lower than the IL containing formulations, highlighting that the conformational transitions are temperature dependant and also conditional on the presence of IL.^10^

This rationalisation is supported by the MD simulations that point to enhanced protein flexibility and greater conformational sampling of IgG4 in water, IL and F1IL at higher temperatures. This is clearly evidenced in F1IL, by an increase in the preferential interactions of arginine with IgG4 and decrease of trehalose interactions with IgG4 as the temperature is increased. This results in increased exposure of hydrophobic residues at the protein surface and alteration of the protein–excipient interactions. Moreover, from simulations of the IL formulation at higher temperatures, the mobility of IgG4 could increase gradually as the molecules continued to explore different conformational environments, due to their inherent flexibility. This is attributed to an increase in preferential binding of water to the polar regions of the protein, coupled with a small increase of IL interactions with hydrophobic residues on the protein. Conversely, for F1, the protein–excipient preferential binding coefficients were essentially constant at both temperatures. Accordingly, the lower protein flexibility and effects of confinement at high temperature explain the barrier to small-scale conformational changes and high *T*_onset_ value determined from CD data, as well as the plateau phase in the region above *T*_m_1__ (Fig. 2).

### Probing the link between matrix–antibody interactions and thermodynamics of conformational transitions

While intramolecular hydrogen-bonding interactions could also stabilise the three-dimensional structure of IgG4, the isolated secondary structures are not stable in solution on their own and the IgG4 chains could adopt different conformations of β-sheets.^5,28,44,48^ We consider that for IgG4 in water, tightly bound water molecules could enhance the flexibility of the antibody, by further increasing the degrees of freedom in the system,^51^ similar to a plasticiser in a polymer. Accordingly, increasing the temperature above *T*_m_2__ likely resulted in more highly denatured or more flexible chain conformations, explaining the high Δ*S*_m_2__ value observed for IgG4 in water (Table 2).^52^ This narrative is supported by the water simulations, where the increased conformational flexibility at high temperatures is coupled with more mobile water molecules that interact with IgG4 *via* short-lived hydrogen bonds. Thus, the mechanism of conformational transitions triggered by temperature is likely similar for F1 and F2, yet different to the antibody in water and the IL containing formulations.

**Fig. 10:**
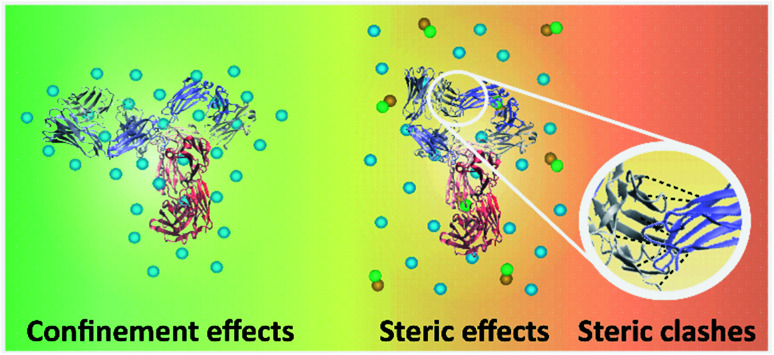
Schematic of the interactions between different parts of IgG4, and IgG4 and the matrix molecules. The three-dimensional structure of IgG4 is illustrated,^44,45^ blue spheres represent the excipient molecules, and green and orange spheres represent the IL molecules. On the left, high-temperature macromolecular confinement effect for F1 and F2 is demonstrated, which could lead to relatively stable residence of conformational sates in wells of the local free energy minima. On the right steric strain in IgG4 structure is demonstrated, which could facilitate conformational motions that help the molecules jump out of the wells.

Immobilising and stabilising proteins in a rigid matrix has been previously shown to slow down thermal degradation.^28,53,54^ It can be suggested that IgG4 was rigidly stabilised by the excipient molecules in F1 and F2, resulting in a relatively high *T*_onset_ and barrier to small-scale conformational changes. Specifically, for F1 the positively charged amino acid l-arginine could bind to the negatively charged surfaces of IgG4 and the guanidinium group could form salt bridges with the antibody acidic residues, thereby stabilising IgG4.^25,32^ Furthermore, the higher *T*_m_1__, Δ*H*_m_1__ and Δ*S*_m_1__ values observed for F1 compared to F2, is explained by considering that due to the higher protein concentration of F1, the extent of crowding or confinement of the protein excipient matrix is increased (Fig. 10), and confinement led to enhanced thermal stability. Thus, we suggest that for F1 and F2, the plateau phase in the region above *T*_m_1__, indicated localised confinement of the protein–excipient matrix, hindering further conformational transitions; and the effect of confinement depended on the protein concentration and excipient matrix.^32,55^

When considering the IL containing formulations, we found that Δ*S*_m_1__ was lowest for IgG4 in IL (910 J K^−1^ mol^−1^) and highest for F2IL (1050 J K^−1^ mol^−1^), suggesting that in the absence of excipients, the antibody was more susceptible to conformational transitions. Specifically, the higher value of Δ*S*_m_2__ than Δ*S*_m_1__ could be due to steric strain on the antibody structure at *T*_m_2__ owing to steric clashes of the β-sheets at higher temperatures (Fig. 10).^56,57^ Based on previous work, it was expected that with heating, the formation of β-sheet interactions would induce conformation changes, which can result in conformational restrictions consequently limiting the number of accessible conformational states.^3,5^ This would explain the relatively small change in secondary structure observed in the second sigmoidal regime. In line with this, the observed decrease in Δ*S*_m_3__ compared to Δ*S*_m_2__, for all IL containing formulations, most prominently for F2IL, suggested that above *T*_I_2__, the IgG4 structure became increasingly unstable, likely due to steric clashes as illustrated in Fig. 10. This would have caused the structure of IgG4 to collapse and lead to the loss of the conformational entropy of the system observed at *T*_m_3__.^57–61^

Analysis of the MD simulations revealed that the excipients were preferentially included at the protein–solvent interface for F1 (Fig. 6A). Specifically, the interactions involved were dominated by the formation of hydrogen bonds between trehalose and the polar residues of IgG4; as well as *via* hydrogen bonding and cation–π interactions between arginine with the polar and hydrophobic regions of IgG4. Thus, the effects of confinement on IgG4 arise from increased non-specific electrostatics interactions and strong hydrogen bonding between the constituent ions and the polar and charged (positive and negative) residues on the protein surface. Notably, these interactions were largely retained at high temperatures, further supporting the observation of high temperature macromolecular confinement in the absence of IL. Moreover, MD simulations showed that at low temperatures, the IL system exhibited similar behaviour to that of F1, consistent with the systems exhibiting molecular confinement of IgG4 in its surrounding matrix below *T*_onset_. It should be highlighted, that for F1IL the system is more complex, and thus the addition of IL led to the formation of large excipient–IL (cosolvent) clusters formed of trehalose, arginine and dihydrogen phosphate (Fig. 6B). As such, these clusters were bound to specific regions of the Fab fragment, forming a diverse protein-cosolvent surface, effectively dampening the flexibility of the protein. On heating (simulations at high temperatures), the hydrogen bonding interactions between the IL and IgG4 were weakened in the IL formulation, and an increased preference for weakly-bound water molecules to the protein surface increased the local structure flexibility. Similarly, for F1IL, the excipient–IL clusters dissociated with heating, and a more even distribution of the excipients was found at the protein surface (Fig. 6C and D). This even distribution of cosolvent molecules, corresponded to the decrease in the preferential binding coefficient for trehalose and increase for arginine. This likely results from the formation of arginine–dihydrogen phosphate ion pairs and clusters that can interact more effectively with the changing protein surface.^62^ Thus, these results suggest that the total free energy change of both IL and F1IL (Table 3 and Fig. 3) is greatly influenced by the dihydrogen phosphate anions.

For each formulation, Δ*G*_total_ was the total free energy change of the antibody-matrix system. The conformational entropy of the antibody, the IgG4–IgG4 and IgG4–matrix interactions would lead to free energy fluctuations,^8,63^ yet such fluctuations were likely too small to cause substantial directional conformational transitions below *T*_onset_. However, heating above *T*_onset_, and further above *T*_m_1__ resulted in a steep gradient (Fig. 3), which could be interpreted as a force, for example hydrogen-bonding interactions, that drove the molecules to search for the free energy minimum.^61^ We propose that the force originated from the matrix, which confined the antibody and directed the system toward the lower free energy conformations.^32^Fig. 3 shows for F1 and F2, the conformers became trapped within a free energy minimum, indicated by the Gibbs free energy change presenting a plateau-like behaviour at high temperatures above *T*_m_1__. For water and the IL containing formulations, above *T*_m_1__ the conformers also became transiently trapped within free energy minima; yet, distinctly, with sufficient energy input, the trapped conformers could jump out of local free energy wells and climb up the steep slope. Fig. 3B clearly showed that increasing the temperature further, at *T*_m_3__ we observed an increase in Δ*G*, along with a decrease in Δ*H*_m_3__ and Δ*S*_m_3__ and correspondingly a decrease in the β-sheet content of the conformers. It can be suggested that a reduction in the entropy gain during denaturation and an increase in the free energy needed for unfolding explains our observed increase in Δ*G* and loss of β-sheet content at higher temperatures,^6,8^ most significantly above *T*_m_3__. Notably, it appeared that for the IL containing formulations, the entropy effect contributed the most to the decrease of Δ*G* for the IgG4–IL system, as highlighted by the case of IgG4 in IL which showed the highest value for Δ*S*_total_ and lowest value for Δ*G*_total_. Thus, we proposed that the temperature dependence of Δ*G* appeared to show a relatively large entropic contribution to the thermostability and conformational stability of IgG4, however this requires further investigation.^64^

### New formulations identified with potential for reviving lost therapeutic candidates

Thus far, we have shown that in distinct formulation environments, the antibodies appeared to use different conformational transition pathways, emphasising that while the amino acid sequence of a protein defines its three-dimensional conformation, the environment dictates the conformational transition path. Our choice of excipients and study of ILs was also motivated by the growing need to identify novel excipients for improving the thermal and structural stability of protein formulations. By conducting BLI experiments we could examine possible trade-offs between IgG4 physical stability and structural alterations affecting its binding ability in the presence of IL and different excipients (Fig. 7). We emphasise that the strongest piece of evidence for ILs impacting the conformations of IgG4 and adversely affecting ligand binding was the increase in *K*_D_ values for all the IL containing formulations. This suggested that in the presence of IL, structural alterations of IgG4 occurred. Moreover, we observed that the excipients in F1 seemed to attenuate the negative effects of the IL on the IgG4–ligand complex formation. This was indicated by the minimal change observed on the sensorgram, and the lower *K*_D_ value obtained compared to that of IL and F2IL, respectively. These observations also supported our earlier notion that the structural stability of IgG4 should be considered in the context of the effects of the matrix, and specific excipients, on the conformational transitions of IgG4. Finally, our results indicated that for F1IL, the binding of IgG4 on both the Fab and Fc regions showed minimal alterations, compared to that observed for IgG4 in water and F2IL, respectively. This suggested that the formulation candidate F1IL preserved IgG4 structure from a functional point of view, and thus [Cho][DHP], trehalose dihydrate, arginine-HCl, and polysorbate 20 were identified as promising stabilisers that could potentially protect antibodies against conformational destabilisation, aggregation, and chemical degradation.

## Conclusions

Exploring the conformational transitions of antibody systems is necessary for understanding the mechanism of protein folding, to gain insight into natural and disease related processes and for biotechnological applications. Herein, we developed a series of unique IL containing formulations and introduced a comprehensive methodology to examine the structural and thermal stability of antibodies in solution, as well as the conformational transitions, energetic properties, and thermodynamic behaviours of complex systems. Variable temperature CD spectroscopy experiments showed secondary structural changes of IgG4 in relation to thermal denaturation, and enabled an experimental conformational search on antibodies, which can be applied to other proteins and biomolecules. Furthermore, the CD analysis aligns with the MD simulations that found that preferential interactions between the excipients and IL and IgG4, drive the protein conformational changes. As well, the thermodynamic parameters, determined from CD experiments, were supported by MD simulations that showed that the barrier to conformational change in IL and F1IL was lowered by the addition of [Cho][DHP], whereas in F1 localised confinement of the protein–excipient matrix hindered conformational changes. Additionally, the effects of confinement on IgG4 arise from increased non-specific electrostatics interactions and strong hydrogen bonding between the constituent ions and the polar and charged residues on the protein surface. Finally, we examined and compared the obtained sensorgrams and rate constants obtained by BLI measurements for each formulation. We identified that formulations, such as F1IL, including sugar, amino acids, surfactant, and IL are promising candidates for additional evaluation on the stability of antibodies with ILs. Notably, the conformational stability and dynamics of the protein can potentially be modulated with judicious selection of excipients and IL. We hope that by combining our experimental, computational and analytical approach we are able to contribute to the literature and provide unique insight into the complex process of protein folding and innovative pharmaceutical development based on the distinctive properties of IL containing formulations.

## Materials and methods

### Antibody production

IgG4 was produced from Chinese Hamster Ovary (CHO) cells, cultured at 37 °C, 140 rpm and 8% CO_2_. Briefly, cells were sub-cultured every three days, using CD CHO medium (Thermo Fisher Scientific Inc., Waltham, Massachusetts, USA) and the samples were taken on the harvest day. To isolate the supernatant containing the IgG4, the samples were centrifuged at 800 g for 5 minutes, and the samples were stored at −20 °C. For IgG4 purification, samples were dialysed against binding buffer (0.01 M disodium phosphate, 0.1 M sodium chloride, pH 7.2) at 4 °C for 24 hours. The samples were centrifuged (4000 rpm) at 4 °C for 30 minutes and the supernatant was filtered with a 0.22 μm PES syringe filter and purified by protein A chromatography (see ESI†).

### Production and characterisation of the ionic liquid and the antibody formulations

Trehalose dihydrate, l-histidine, l-histidine-HCl, l-arginine HCl, polysorbate 20 (poly-oxyethylene sorbitan monooleate), choline dihydrogen phosphate ([Cho][DHP]) were all purchased from Sigma-Aldrich Company Limited (Gillingham, Dorset, UK), and stored as recommended. Ultrapure water was used for preparing the IL and formulations, obtained from a PURELAB Ultra water purifier (ELGA LabWater, High Wycombe, UK) with resistivity of 18.2 MΩ. An aqueous stock solution of choline dihydrogen phosphate ([Cho][DHP]) was prepared by addition of ultrapure water to the salt, then shaking at room temperature for 1 hour to give 80 wt% IL solution. The IL was characterised by ^1^H and ^13^C NMR spectroscopy in D2O at 25 °C (Fig. S3†) using Bruker Avance III, 400 MHz spectrometer (Bruker Corporation, Billerica, Massachusetts, USA).

Two excipient stocks were prepared. Solution 1 contained: l-arginine HCl (34 mg mL^−1^), trehalose dihydrate (50 mg mL^−1^), polysorbate 20 (0.49 mg mL^−1^). Solution 2 contained: trehalose dihydrate (25 mg mL^−1^), polysorbate 20 (0.20 mg mL^−1^), l-histidine (0.53 mg mL^−1^), l-histidine HCl (2.2 mg mL^−1^). The formulations were prepared as follows. The water formulation contained 100 mg mL^−1^ of IgG4 in ultrapure water and the IL formulation contained 100 mg mL^−1^ of IgG4 in solution of ultrapure water with 10 wt% [Cho][DHP] from the 80 wt% stock solution. F1 and F2 were prepared by the addition of 100 mg mL^−1^ and 50 mg mL^−1^ of IgG4 to solution 1 and solution 2, respectively. To produce F1IL and F2IL, [Cho][DHP] was added to samples of F1 and F2 to a concentration of 10 wt% (see Table S2† for a tabulated version of these component mixtures). In all formulations the pH range was measured to be between 5.5 and 5.7. Solutions were filtered using 0.22 μm syringe filter units, and the ultraviolet-visible (UV) absorbance at 280 nm (UV-Vis spectrophotometer, Thermo Fisher Scientific Inc., Waltham, Massachusetts, USA) was checked to confirm that the desired concentration of IgG4 in each formulation was achieved (Fig. S4†). All formulations were prepared, immediately stored at 4 °C and measured.

### Variable temperature circular dichroism (CD) experiments

To examine the structure and determine the thermal stability of the formulations, temperature variable CD experiments were performed on a Chirascan CD spectrometer (Applied Photophysics Ltd, Leatherhead, Surrey, UK) in combination with a Quantum Northwest Peltier temperature controller (Quantum Northwest Inc., Liberty Lake, Washington, USA). Each sample was placed in a quartz cuvette (Starna Scientific Ltd, Ilford, UK) and measurements were obtained with a heating rate of 1 °C min^−1^ between 25 °C and 97 °C with a tolerance of 0.2 °C. Spectroscopic data was collected with 1 nm steps between 260 and 190 nm, with 2 seconds collection time per step. For each matrix, background spectra were recorded in triplicate at room temperature, averaged and subtracted from the temperature variable CD data of the respective IgG4 formulation. The Origin software (OriginLab Corporation, Northampton, Massachusetts, USA) was used to analyse the spectroscopic data. The data was zeroed between the 250–260 nm region and smoothed using Savitzky–Golay method with 7 points and polynomial order 5. The mean residue ellipticity (MRE) was calculated by accurately accounting for antibody size and concentration in each sample.

### Relative fraction of β-sheet content determination

The CD data at 218 nm was converted into a plot of the relative fraction of β-sheet content, *f*_β-sheet content_, of the protein at each temperature. For sigmoidal data, the relative change in β-sheet content was treated as a two-state equilibrium model. By establishing a linear dependence of CD signal on the temperature at low *T*, we calculated *y*_0_*,* the predicted CD signal of the IgG4 conformers with minimum β-sheet content at temperature *T* using^65^

1 *y*_0_ = *a*_0_ + *b*_0_*T*

with *a*_0_ and *b*_0_ as temperature-independent constants. The equivalent linear relationship for *y*_max_, the predicted CD signal of the conformers with maximum β-sheet content, and *T* was derived, with *a*_max_ and *b*_max_ calculated from a linear fit at high *T* values. From here *f*_β-sheet content_ was calculated by logistic regression as2
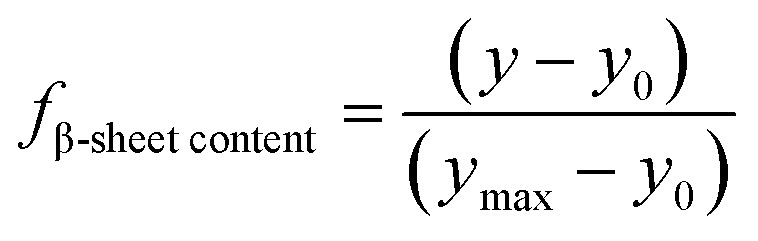
where *y* is the experimentally measured CD signal. Here, *y*_0_ and *y*_max_ represent the minimum and maximum of β-sheet content, with *f*_β-sheet content_ = 0 at *y* = *y*_0_ and *f*_β-sheet content_ = 1 at *y* = *y*_max_. A Boltzmann exponential curve was fitted to the *f*_β-sheet content_ data.

For formulations exhibiting different behaviour, *f*_β-sheet content_ content was estimated using the same formula, but taking *y*_0_ as the minimum magnitude CD signal (the endpoint) and *y*_max_ as the strongest CD signal (the maximum of the negative peak).

Parabolic, Gaussian and bi-Gaussian (an asymmetric Gaussian) fits, to guide the eye, were trialled on the *f*_β-sheet content_ data, with the root mean square error (RMSE) for each fit calculated by3
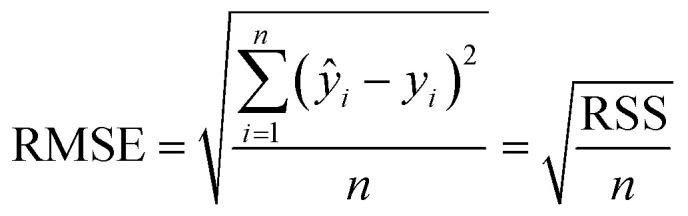
with *n* the total number of points to be fitted, *yŷ*_1_, …, *yŷ*_*n*_ the predicted values, *y*_1_, …, *y*_*n*_ the observed values, and the residual sum of squares (RSS), as calculated by the Origin fitting. In each case the bi-Gaussian fit had the minimum RMSE, so was chosen as most suitable.

### Determining transition temperatures and extracting thermodynamic parameters

A best fit analysis for each data set was developed to establish the number of regions as well as the corresponding transition points between the different regimes. We have based our analysis on experimental data in order to obtain the midpoints, *T*_m_, and the transition points, *T*_I_, on a Gaussian relationship. *m* linear plateau regions were identified for each data set, with data points included to maximise the *R*-squared value of the linear fit. *r* sigmoids were fitted, with one between each sequential pair of linear regions inclusively and the final sigmoid fitted between the *m*th linear region and the end point of the data, the minimum point of the Gaussian curve. Within the Gaussian curves, we observed at least two, and in most cases three, sigmoid regions in the temperature range studied (*r* = 2 or 3), with characteristic intersections between them. The transition temperature between the *i*th and (*i* + 1)th regimes, *T*_I_i__, was taken as the intersection of two neighbouring sigmoidal regions, was calculated as the temperature at which the β-sheet content value of the two sigmoids was equal.

The thermodynamics of the transition between the different conformations present were calculated using the two-stage model described for the sigmoidal curves. For a data set with *r* sigmoids fitted, each sigmoidal region was treated as a separate two-stage equilibrium transition and a logistic regression model applied to each. An equivalent *y*_0_*i*__ and *y*_max_*i*__ were calculated as the initial and final conformations of the protein within the *i*th region as in eqn (1). The fraction of protein in the final conformations of each 2-stage equilibrium was then calculated as in eqn (2), with the rest assumed to be in the initial conformations.

From this fraction, the equilibrium constant of the *i*th region could be determined as4
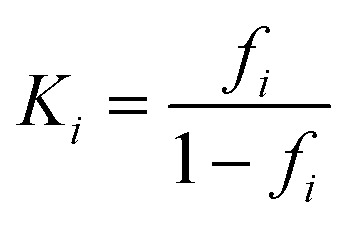
and from there, the free energy of transition in the *i*th regime, Δ*G*_m_*i*__, was calculated as

5 Δ*G*_m_*i*__ = −*RT* ln *K*_*i*_

for ideal gas constant *R*. Δ*G*_m_*i*__ can be plotted linearly against temperature in the transition region (−5 kJ mol^−1^ < Δ*G*_m_ < 5 kJ mol^−1^), which describes the behaviour of Δ*G*_m_*i*__ around the equilibrium temperature between the *i*th and (*i* + 1)th states (*T*_m_*i*__), representing the point at which exactly half of the protein is the initial and final ensembles, respectively and Δ*G*_m_*i*__ = 0. *T*_m_*i*__ also corresponds to the midpoint of the *i*th sigmoid. The enthalpy (Δ*H*_m_*i*__) and entropy (Δ*S*_m_*i*__) of each transition can also be estimated from the Δ*G*_m_*i*__*vs. T* plot using

6 Δ*G*_m_*i*__ = 0 = Δ*H*_m_*i*__ − *T*_m_*i*__Δ*S*_m_*i*__

### Modelling of Fab fragment and excipients

The structure of the chimeric Fab' fragment of the IgG4 antibody was modelled based on the crystal structure (resolution 3.2 Å, PDB entry: 1BBJ).^66^ The structural model included all of the residues of chain A (light chain). Similarly, for chain B (heavy chain) all residues were included apart from the non-standard residue pyroglutamic acid (PCA), which was substituted for glutamine (GLN). Four disulfide bonds were added. Two in the variable region, between CYS23 and CYS88 in VL and between CYS22 and CYS96 in VH (variable heavy). The other two disulfide bonds are in the constant regions, between CYS134 and CYS 194, and CYS141 and CYS197.

Protonation states were assigned at the experimental system pH of 5.5 using the H++ webserver.^67^ Using this method an overall charge of +8*e* was assigned to the Fab fragment. To account for potential local changes in the protonation state (at lower pH) of the protein in IL containing systems, the protonation states of the Fab fragment were determined at pH 4.5 and 5.0. The overall charge was found to remain at +8*e* at both these additional pH values.

For simulations using explicit water, the protein was solvated in a cubic TIP3P water box with a minimal distance of 14 Å between the boundaries of the box and the nearest protein atoms using the tleap module in Ambertools18.^68^ This added 36 571 solvent water molecules. Cl^−^ anions were added to neutralise the charge. For simulations including the IL and excipients, the number of species of the respective additives was determined based on the experimental concentrations. The starting structures for the IL, F1 and F1IL simulations were generated using the Packmol software^69^ and parameter and topology files were generated using tleap. A summary of the number of ions and excipients included is provided in the ESI, Table S4.†

### Molecular dynamics simulations and analysis

The solvated structures were all first subjected to a two-stage energy minimisation. In the first stage of 5000 steps, the protein was restrained to its crystallographic positions using a harmonic potential with a force constant of 25 kcal mol^−1^ Å^−2^ while all other atoms were unrestrained. In the second stage of 5000 steps, no restraints were applied. In both stages the steepest descent method was used for the 1st 2500 steps and the conjugate gradient method was switched on for the rest of the steps. Each system was then subjected to 500 ps of heating (NVT) and 1 ns pre-equilibration in the NPT ensemble to obtain the required temperature and density. Three independent 100 ns simulations at 27 °C (300 K) and two independent 200 ns simulations at 127 °C (400 K) were then carried out for each system in the NPT ensemble.

The parameters for protein residues were assigned based on the Amber ff14SB force field.^70^ Parameters for trehalose were assigned using Glycam06-j.^71^ Amber consistent parameters were employed for arginine cations^72^ and chloride anions.^73^ The GAFF (General Amber Force Field) was used to describe the polysorbate 20 and IL (choline and dihydrogen phosphate) species,^74^ with unity charges assigned using the RESP method at the HF/6-31g* level of theory.^75^

All simulations were carried out using Amber18.^68^ Temperature and pressure were controlled by employing the Langevin thermostat (collision frequency of 1 ps) and the Berendsen barostat (1 atm, relaxation time of 1 ps), respectively.^76,77^ All bonds involving hydrogen atoms were constrained using the SHAKE algorithm.^78^ Long-range electrostatic interactions were accounted for by using Particle Mesh Ewald method,^79^ and a non-bonded cut-off of 12 Å was applied. A 2 fs time step was employed and all simulation trajectories were saved every 10 ps.

Primary analysis of each trajectory was carried out with CPPTRAJ.^80^ The preferential binding coefficients were determined employing the approach and software (ComplexMixtures.jl – v0.4.13) developed by Martínez using *R* = 12 Å.^81^

### Biolayer interferometry (BLI) measurements and analysis

BLI experiments were performed with a BLItz® (Sartorius AG, Göttingen, Germany) interferometer using AHQ Anti-Human IgG Fc Specific Biosensors, product number 18-5001 and AMC Anti-Murine IgG Fv Biosensors, product number 18-5022 (FortéBio, Fremont, California, USA), for analysis of the antigen-binding fragment (Fab) and the fragment crystallisable (Fc). The sensors were hydrated prior to use by submerging them on sample diluent (Sartorius AG, Göttingen, Germany) for at least 10 minutes, and kept submerged in the buffer until use, for a maximum of 1 hour. The BLI assays were composed of three steps: (i) equilibration of the sensor for 1 minute in sample diluent, (ii) IgG4 adsorption step by loading 4 μL of antibody at 0.96 mg mL^−1^, diluted in sample diluent, for 2 minutes, and (iii) desorption step on a 250 μL sample of the correspondent formulation used in step (ii) without IgG4. The sensorgrams obtained were fitted considering a 1 : 2 binding model with the R pbm package (https://github.com/jonathanrd/pbm) in order to obtain the binding kinetic parameters (see ESI† for details on the estimation of 1 : 2 and 1 : 1 binding models parameters). Simulations were run in RStudio version 1.2.5042 using R 4.0.0. The fitting parameters were the sets of *k*_on_, *k*_off_ and *R*_max_ for the first and second binding and dissociation phases using7
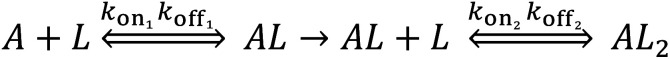
where *A* is the analyte (IgG4), *L* is the monovalent ligand, *AL* is the intermediate complex with one ligand molecule and *AL*_2_ is the final complex with two ligand molecules and both of the binding sites of the analyte occupied. *k*_on_ (M^−1^ s^−1^) and *k*_off_ (s^−1^) are identified as the association and dissociation rate constants, respectively, and *R*_max_ (nm) represents the highest binding signal. *k*_on_ describes the rate of formation of the bound analyte–ligand complex while *k*_off_ represents the fraction of analyte–ligand complexes that decay per unit of time and is related to the stability of that complex. *K*_D_ (M) is the analyte concentration at which 50% of the ligand sites are occupied at equilibrium. Thus, the lower the *K*_D_ value, the tighter the interaction between the ligand and the analyte. *K*_D_ was calculated using8
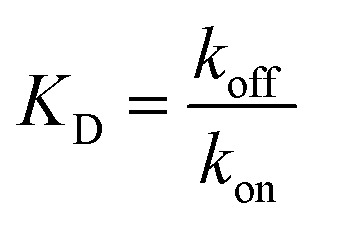


## Data availability

All data needed to evaluate the conclusions in the paper are present in the paper and/or the supplementary materials. Additional data related to this paper may be requested from the authors.

## Author contributions

T. A. S. conceived the project, designed the formulations, performed the circular dichroism (CD) and BLI experiments, analysed the data and wrote the paper with L. K. M., I. M.-R., R. P. M. and P. K.; L. K. M. and T. A. S. developed the method for CD data analysis; L. K. M. prepared the CD figures and tables and schematics and L. K. M. and T. A. S. analysed the CD data; I. M.-R. and P. K. prepared the BLI figures and tables and analysed the BLI data; L. B.-L. purified the protein, prepared the formulations with T. A. S. and performed some of the CD and BLI experiments with T. A. S.; R. P. M. and G. A. V. performed and analysed the molecular dynamics simulations data; L. K. M., I. M.-R., P. K., R. P. M. and T.A.S. prepared the Supplementary Materials. J. P. H. supervised the research and directed the project with T. A. S.; J. P. H., K. M. P. and C. K. assisted with framing and development of ideas; and all authors provided comments and approved the manuscript.

## Conflicts of interest

T. A. Shmool and J. P. Hallett, UK Pat., 2108009.8, 2021. Other authors have no conflicts to declare.

## Supplementary Material

SC-012-D1SC02520A-s001
