# Supplementary material for: An experimental approach probing the conformational transitions and energy landscape of antibodies: a glimmer of hope for reviving lost therapeutic candidates using ionic liquid
Source: Chem Sci. 2021 Jun 22;12(27):9528–45. doi: 10.1039/d1sc02520a (PMC8278930; doi:10.1039/d1sc02520a)
Supplement: SC-012-D1SC02520A-s001 [file SC-012-D1SC02520A-s001.pdf]

This journal is © The Royal Society of Chemistry 2021

An Experimental Approach Probing the Conformational Transitions and Energy Landscape of Antibodies: A Glimmer of Hope for Reviving Lost Therapeutic Candidates Using Ionic Liquid

Talia A. Shmool[1], Laura K. Martin[2], Liem Bui-Le[1], Ignacio Moya-Ramirez[1], Pavlos Kotidis[1], Richard P. Matthews[1], Gerhard A. Venter[3], Cleo Kontoravdi[1], Karen M. Polizzi[1] and Jason P. Hallett[1,*]

[1] Department of Chemical Engineering, Imperial College London, South Kensington Campus, London SW7 2AZ, UK.
[2] Department of Engineering Science, University of Oxford, Parks Road, Oxford OX1 3PJ, UK.
[3] Scientific Computing Research Unit and Department of Chemistry, University of Cape Town, Rondebosch, Cape Town 7701, South Africa.
[*] Corresponding author. Email: j.hallett@imperial.ac.uk. Tel.: +44 (0)20 7594 5388

Immunoglobin G4 (IgG4) purification

Dialysed IgG4 solution was purified by protein A chromatography using two HiTrap MabSelect SuRe pcc (Cytiva, formerly GE Healthcare Life Sciences, Uppsala, Sweden) columns placed in series and mounted onto an ÄKTApurifier 900 (GE Healthcare Bio-Sciences AB, Uppsala, Sweden), using a flow rate of 1 mL/minute. Binding buffer (0.01 M disodium phosphate, 0.1 M sodium chloride, pH 7.2) was used to equilibrate the columns with 10 column volumes (CV). Once equilibrated, the IgG4 sample was passed through the columns. Ten CV of binding buffer was injected into the column, and the absorbance at 280 nm was used to detect the protein content. Elution buffer (0.01 M disodium phosphate, pH 3) was used to elute IgG4 into aliquots of 1 CV, that were supplemented 0.02 CV of neutralising buffer (0.5 M disodium phosphate, pH 9.0) after elution. Once the total protein content was negligible, 20 CV of binding buffer was passed through to equilibrate the columns before repeating the purification cycle. IgG4 aliquots were combined and concentrated using an Amicon Ultra 50 kDa Centrifugal Filters spin filter (Merck KGaA, Darmstadt, Germany) to a concentration of 2 mg/mL. The samples were freeze-dried and stored at -20 °C. Protein A columns were stored at 4 °C in 20 % aqueous ethanol for less than a month or in absolute ethanol for long-term storage.

Binding model fitting

For both the 1:2 and 1:1 binding models analysis, the R pmb package (https://github.com/jonathanrd/pbm) was utilised. The "*binding2to1*" and "*binding1to1*" functions were used for the regression of a 1:2 and 1:1 binding model, respectively, and the

estimation of the model constants (k_{on2}, k_{off2} and R_{max}). The analysis was performed in RStudio version 1.2.5042 using R 4.0.0 (RStudio, Boston, Massachusetts, USA). The analyte concentration was set at 6×10^{-6} M and remained constant, as it was common for all experiments. The time point for the initiation of the dissociation phase (t_d) was set at 120 seconds and the initial values of the estimated parameters were set as following:

$$k_{on1} = 7 \times 10^{4}\ M^{-1}s^{-1}$$
$$k_{on2} = 9 \times 10^{3}\ M^{-1}s^{-1}$$
$$k_{off1} = 1 \times 10^{-2}\ s^{-1}$$
$$k_{off2} = 4 \times 10^{-3}\ s^{-1}$$
$$R_{max1} = 3 \times 10^{-1}\ nm$$
$$R_{max2} = 3 \times 10^{-1}\ nm$$

The k_{on2}, k_{off2} and R_{max2} are only applicable to the 1:2 binding model and "*binding2to1" function.* Instructions on how to install and execute the package in R can be found in the associated online directory.

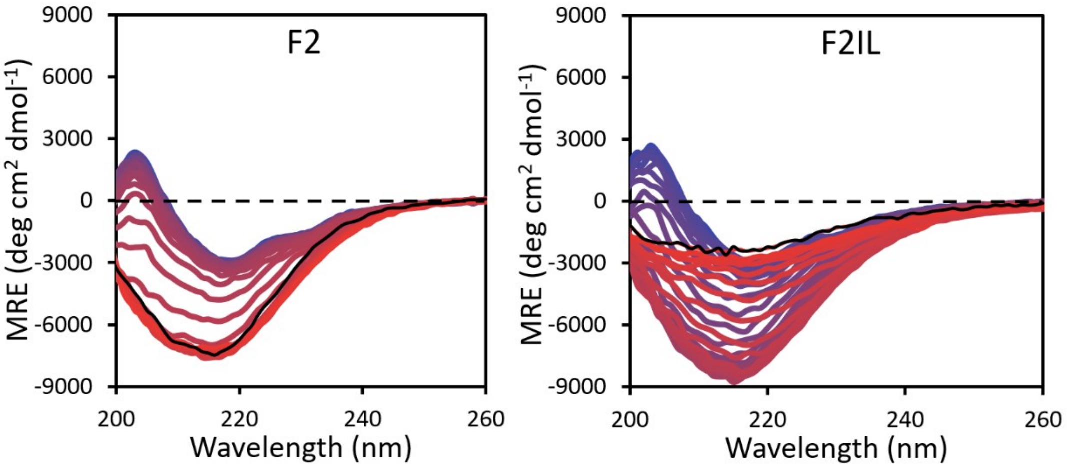

Figure S1: The mean residue ellipticity (MRE) calculated from the temperature variable CD data for F2 and F2IL, measured from 190 to 260 nm with temperature increasing from 25 °C (blue) to 97 °C (red) in 2 °C increments. The reversibility of the structural changes the of IgG4 was examined by cooling F2 and F2IL from 97 °C to 25 °C at 2 °C/min and measuring the CD spectra at 25 °C (black).

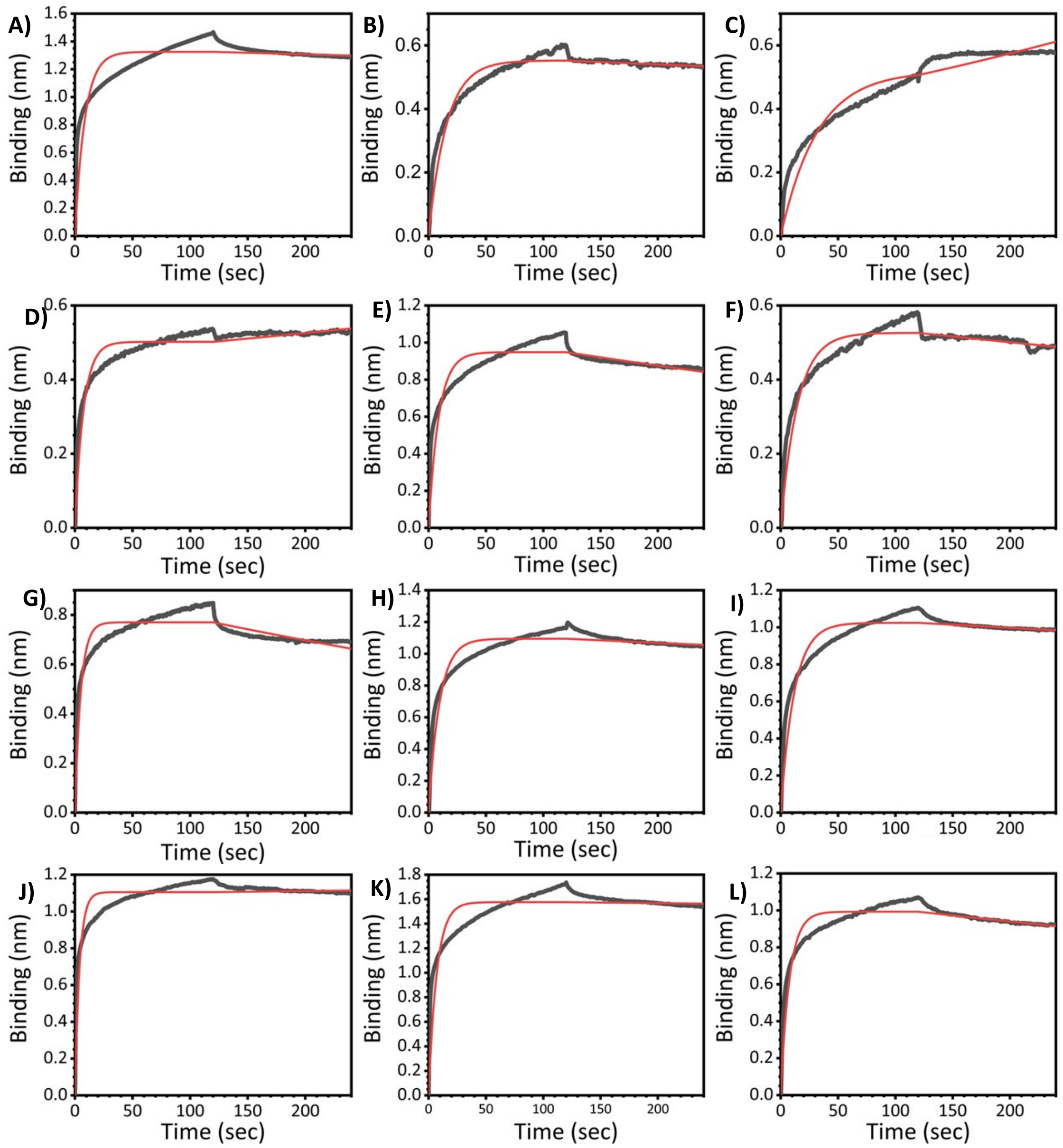

Figure S2: Fitting of the 1:1 binding model for **A)** Fab-F1IL, **B)** Fab-F2IL, **C)** Fab-F1, **D)** Fab-F2, **E)** Fab-IL, Fab **F)** -Water, **G)** Fc-F1IL, **H)** Fc-F2IL, **I)** Fc-F1, Fc- **J)** F2, **K)** Fab-IL, **L)** Fc-Water. After establishing that a 1:1 model did not satisfactorily fit our experimental results, as can be observed in the discrepancies between experimental and fitted data the binding profiles of the IgG4 were fitted to a 1:2 binding model (Figure 7).

Table S1: Constants of the IgG4 fragments binding for each experiment. The binding of the Fab fragment in F2IL could not be fitted and was omitted from the analysis.

Fragment	Formulation	Ionic Liquid	k_{on1} ($M^{-1} \cdot s^{-1}$)	k_{off1} (s^{-1})	K_{D1} (μM)
Fab	Sample diluent	Yes	3.81×10^{4}	6.71×10^{-3}	1.76×10^{-1}
Fc			5.63×10^{4}	6.19×10^{-3}	1.10×10^{-1}
Fab	F1	Yes	4.54×10^{4}	7.13×10^{-3}	1.57×10^{-1}
Fc			4.93×10^{4}	4.44×10^{-3}	9.00×10^{-2}
Fab	F2	Yes	N/A	N/A	N/A
Fc			5.12×10^{4}	6.85×10^{-3}	1.34×10^{-1}
Fab	Sample diluent	No	1.14×10^{5}	1.04×10^{-2}	9.15×10^{-2}
Fc			1.20×10^{5}	6.31×10^{-3}	5.25×10^{-2}
Fab	F1	No	6.40×10^{4}	1.70×10^{-3}	2.65×10^{-2}
Fc			1.29×10^{5}	2.60×10^{-3}	2.01×10^{-2}
Fab	F2	No	1.16×10^{5}	1.10×10^{-2}	9.44×10^{-2}
Fc			1.31×10^{5}	4.75×10^{-3}	3.62×10^{-2}

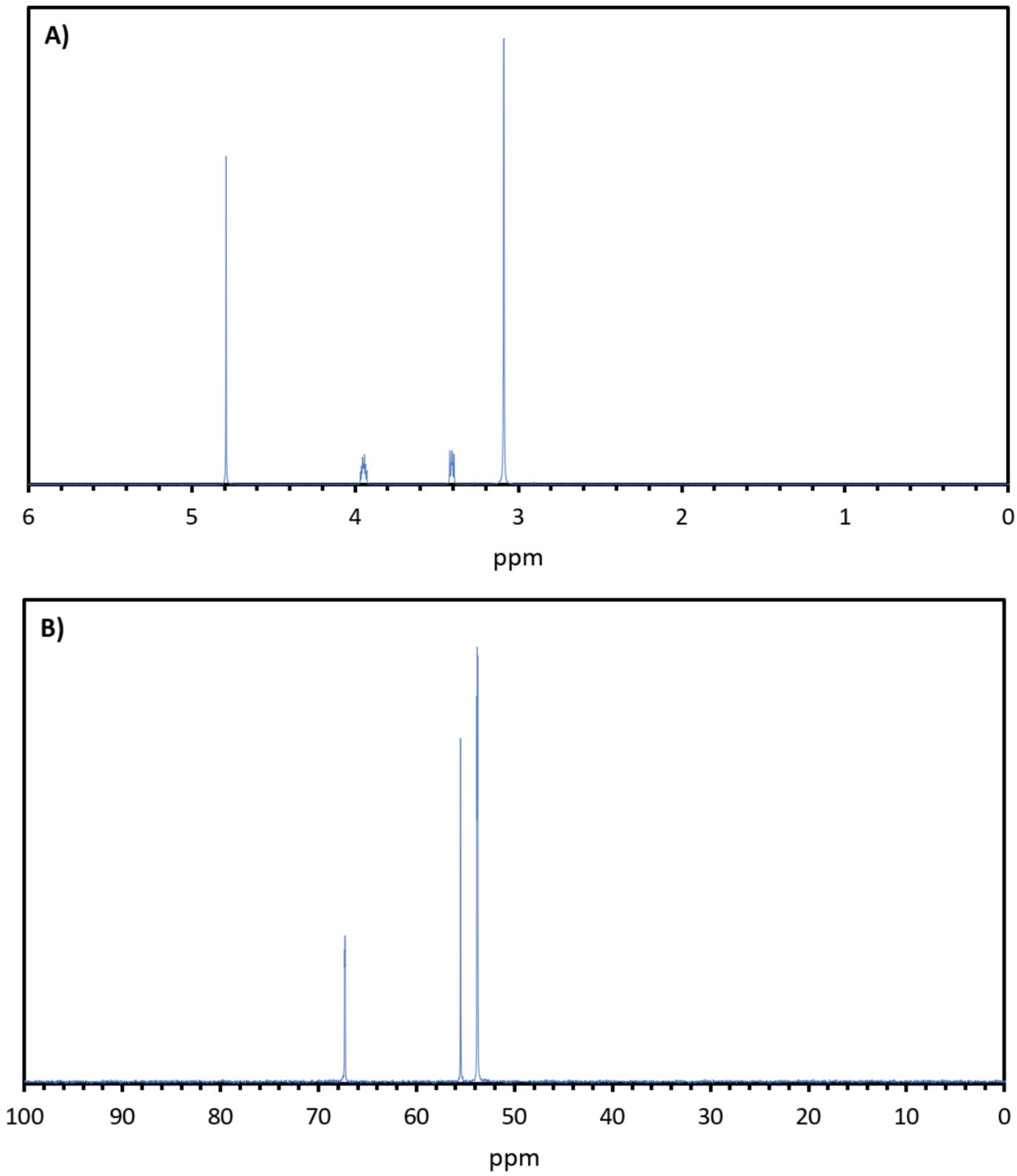

Figure S3: The **A)** ^{1}H and **B)** ^{13}C NMR spectra of [Cho][DHP] used in the IL formulations, measured in D_2O.

Table S2: The composition of the different IgG4 formulations prepared and examined in this work. All formulations were prepared in ultrapure water with the respective components listed.

**Name**	**IgG4 concentration (mg mL^{-1})**	**[Cho][DHP] content (wt %)**	**Components**	**Concentration (mg mL^{-1})**
Water	100	0	-	-
IL	10	10	-	-
F1	100	0	L-arginine HCl<br>trehalose dihydrate<br>polysorbate 20	34<br>50<br>0.49
F2	50	0	L-histidine<br>L-histidine HCl<br>trehalose dihydrate<br>polysorbate 20	0.53<br>2.2<br>25<br>0.20
F1IL	100	10	L-arginine HCl<br>trehalose dihydrate<br>polysorbate 20	34<br>50<br>0.49
F2IL	50	10	L-histidine<br>L-histidine HCl<br>trehalose dihydrate<br>polysorbate 20	0.53<br>2.2<br>25<br>0.20

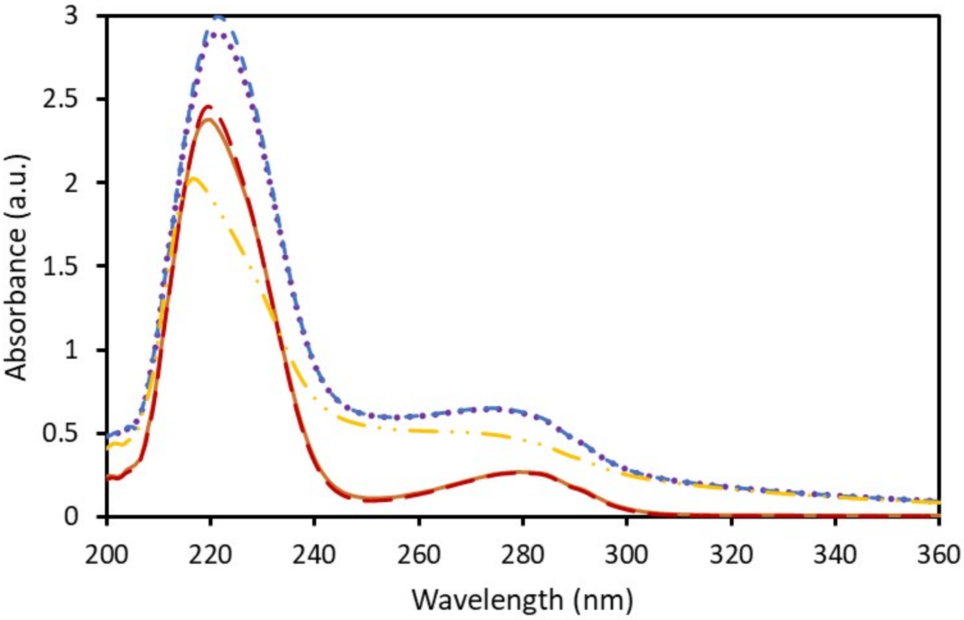

Figure S4: UV-vis spectra of IgG4 in F1 (solid orange line), F2 (dashed red line), 10 wt% [Chol][DHP] (dotted/dashed yellow line), F1IL (dotted purple line), F2IL (small dashed blue line).

Table S3: Preferential interaction coefficients for trehalose, arginine HCl and [Cho][DHP] as a function of concentration (mol.cm^{-3}) in the respective formulations at 27 ºC (300 K) and 127 ºC (400 K). For F1IL, binding coefficients for alternative combinations of [Arginine][DHP] and [Cho][Cl] were also estimated.

		**27 ºC**			**127 ºC**		
		Γ_{MD}	Γ_c	Γ_a	Γ_{MD}	Γ_c	Γ_a
F1	Trehalose	36.1	-	-	37	-	-
	Arginine HCl	5.2	10.6	-0.2	4.1	7.0	1.3
IL	[Cho][DHP]	24.5	20.8	28.8	13.6	10.6	16.8
F1IL	Trehalose	35.5	-	-	28.8	-	-
	Arginine HCl	3.2	8.8	-2.5	9.5	20.6	-1.7
	Arginine DHP	11.3	8.8	45.3	15.9	20.6	36.6
	[Cho][Cl]	4.5	17.2	-2.5	1.5	8.5	-1.7
	[Cho][DHP]	31.3	17.2	45.3	22.6	8.5	36.6

Table S4: Number of each species included in the molecular dynamics simulations. These numbers correspond to the concentrations listed in Table S2.

Simulation	Components	Number of species
Water	Water<br>Chloride anions	36 571<br>8
IL	Water<br>$[Cho]^{+}$<br>$[DHP]^{-}$	36 000<br>355<br>363 (8 extra anions to neutralize system charge)
F1	Water<br>L-arginine HCl<br>Trehalose dihydrate<br>polysorbate 20	36 000<br>108/116 Cl^{-}<br>100<br>1
F1IL	Water<br>$[Cho]^{+}$<br>$[DHP]^{-}$<br>L-arginine HCl<br>Trehalose dihydrate<br>Polysorbate 20	36 000<br>355<br>355<br>108/116 Cl^{-}<br>100<br>1

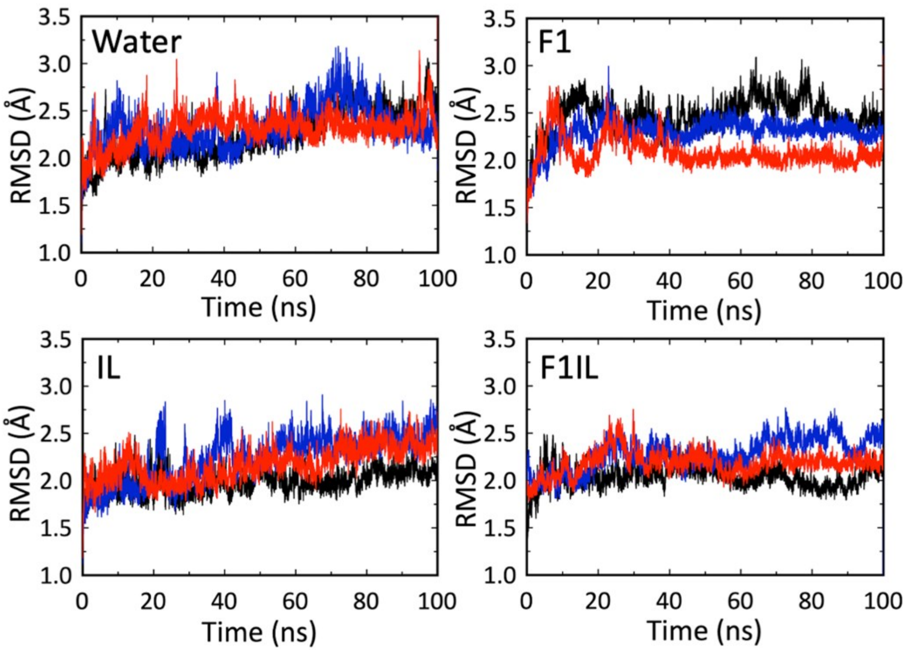

Figure S5: Root-mean-square deviations (RMSD) of the Fab heavy atoms from the X-ray structure during the MD simulations at 27 °C (300 K). The black, blue and red plots are for simulations 1,2 and 3 respectively.

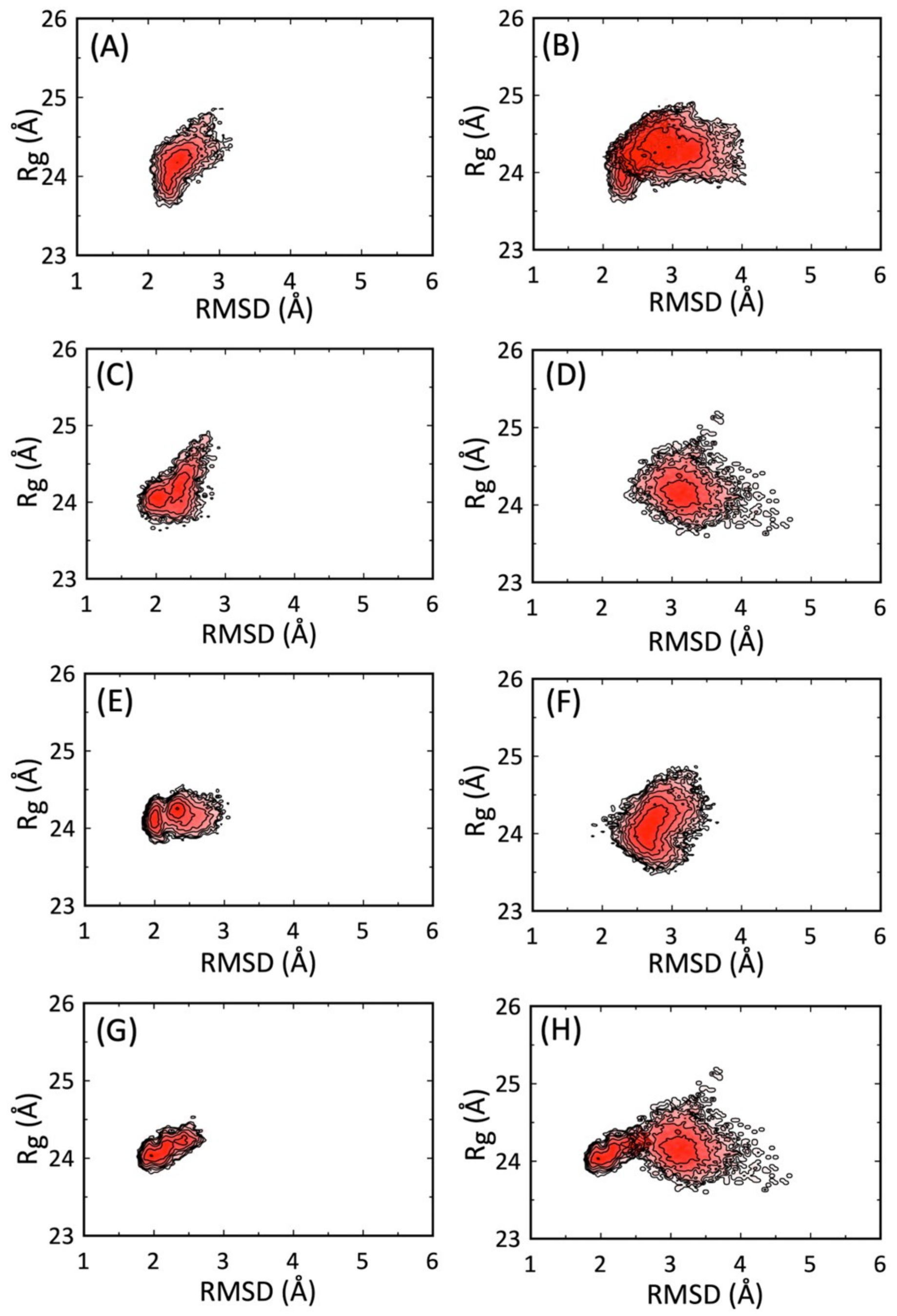

Figure S6: Plots of RMSD vs radius of gyration (R_g) (Å) for the Fab fragment in each formulation at 27 °C (300 K) and 127 °C (400 K). The plots reveal the degree of conformational sampling for the Fab fragment in (A) water, (C) IL, (E) F1, and (G) F1IL at 27 °C, and (B) water, (D) IL, (F) F1, and (H) F1IL at 127 °C.
